# Umbilical cord mesenchymal stromal cells transplantation delays the onset of hyperglycemia in the RIP-B7.1 mouse model of experimental autoimmune diabetes through multiple immunosuppressive and anti-inflammatory responses

**DOI:** 10.3389/fcell.2023.1089817

**Published:** 2023-02-15

**Authors:** C. C. Lachaud, N. Cobo-Vuilleumier, E. Fuente-Martin, I. Diaz, E. Andreu, G. M. Cahuana, J. R. Tejedo, A. Hmadcha, B. R. Gauthier, B. Soria

**Affiliations:** ^1^ Department of Cell Therapy and Regeneration, Andalusian Center of Molecular Biology and Regenerative Medicine-CABIMER, Junta de Andalucía-University of Pablo de Olavide-University of Seville-CSIC, Seville, Spain; ^2^ Institute of Bioengineering and Health Research Institute (ISABIAL), Dr. Balmis University Hospital (HGUA), Miguel Hernández University School of Medicine, Alicante, Spain; ^3^ Department of Applied Physics, University Miguel Hernández, Alicante, Spain; ^4^ Biomedical Research Network on Diabetes and Related Metabolic Diseases (CIBERDEM), Institute of Health Carlos III, Madrid, Spain; ^5^ Department of Molecular Biology and Biochemical Engineering, Universidad Pablo de Olavide, Seville, Spain; ^6^ Instituto de Investigación Biosanitaria, Universidad Internacional de Valencia (VIU), Valencia, Spain

**Keywords:** experimental autoimmune diabetes, intraperitoneal transplantation, inflammation, immunomodulation, insulitis, Type I diabetes mellitus, RIP-B7.1, UC-MSC

## Abstract

Type 1 diabetes mellitus (T1DM) is an autoimmune disorder specifically targeting pancreatic islet beta cells. Despite many efforts focused on identifying new therapies able to counteract this autoimmune attack and/or stimulate beta cells regeneration, TD1M remains without effective clinical treatments providing no clear advantages over the conventional treatment with insulin. We previously postulated that both the inflammatory and immune responses and beta cell survival/regeneration must be simultaneously targeted to blunt the progression of disease. Umbilical cord-derived mesenchymal stromal cells (UC-MSC) exhibit anti-inflammatory, trophic, immunomodulatory and regenerative properties and have shown some beneficial yet controversial effects in clinical trials for T1DM. In order to clarify conflicting results, we herein dissected the cellular and molecular events derived from UC-MSC intraperitoneal administration (i.p.) in the RIP-B7.1 mouse model of experimental autoimmune diabetes. Intraperitoneal (i.p.) transplantation of heterologous mouse UC-MSC delayed the onset of diabetes in RIP-B7.1 mice. Importantly, UC-MSC i. p. transplantation led to a strong peritoneal recruitment of myeloid-derived suppressor cells (MDSC) followed by multiple T-, B- and myeloid cells immunosuppressive responses in peritoneal fluid cells, spleen, pancreatic lymph nodes and the pancreas, which displayed significantly reduced insulitis and pancreatic infiltration of T and B Cells and pro-inflammatory macrophages. Altogether, these results suggest that UC-MSC i. p. transplantation can block or delay the development of hyperglycemia through suppression of inflammation and the immune attack.

## Introduction

Type I diabetes Mellitus (T1DM) is an autoimmune disorder targeting the destruction of pancreatic insulin-producing beta cells, ultimately leading to hyperglycemia and the requirement of insulin therapy for survival. Latest findings indicate that T1DM originates from an early low chronic inflammation and disease of beta cells followed by the collapse of immune tolerance to pancreatic beta cell self-antigens such as insulin in individuals, a process which is facilitated by genetic predisposition and environmental factors (Pociot and Lernmark 2016; Antonela et al. 2017; Jerram et al. 2017; Norris et al. 2020; Roep et al. 2021; von Scholten et al. 2021). Although autoreactive CD45^+^, CD8^+^, CD3^+^ and CD4^+^ T Cells are the main cellular effectors of beta cells destruction (Willcox et al. 2009; Sarikonda et al. 2014), increasing evidence also demonstrated the critical participation of other immune cell types, mainly CD20^+^ B lymphocytes and pro-inflammatory CD68^+^ macrophages in the initiation and progression of the disease (Gaglia et al. 2011; Morgan et al. 2014; Campbell-Thompson et al. 2016; Christie 2016).

Despite advances in our understanding of T1DM progression that has highlighted a relevant number of targets that upon modulation could either prevent or revert hyperglycemia in the Non-Obese Diabetic (NOD) mouse model of T1DM, translation to human patients has been mitigated by poor clinical trial outcomes (Thayer et al. 2010; Warshauer et al. 2020; von Scholten et al. 2021). As such, trials using anti-CD3 or anti-CD20 monoclonal antibodies to inhibit the development and mobilization of either T- or B- cells, led to a mild and transient improvement in C-peptide responses but failed to impede the recurrence of β-cell autoimmunity (Bluestone et al. 2010; Aronson et al. 2014; Vudattu and Herold 2014). A more recent clinical trial using Verapamil, a calcium channel blocker that improves beta cell survival was also shown to improve C-peptide response after a mixed meal highlighting the importance of targeting the metabolic damage to the beta cell mass in addition to the immune cells (Ovalle et al. 2018).

The shortfalls of these “targeted monotherapies” have been a matter of debate and challenge the strategy of whether blocking a specific immune cell type or molecular mechanism is sufficient enough to attenuate the ongoing autoimmune attack and preserve the beta cell mass in T1DM (Cobo-Vuilleumier et al. 2018; Cobo-Vuilleumier and Gauthier 2020). Advanced therapies that simultaneously target multiple key immune events while establishing a local pancreatic anti-inflammatory and regenerative milieu have come into the limelight as a powerful alternative approach for the treatment of T1DM (Canibano-Hernandez et al. 2018; Warshauer et al. 2020). As such, cell-based immunotherapies using *ex vivo* expanded autologous tolerogenic dendritic cells or regulatory T Cells (Tregs) were shown to convey pleiotropic positive effects in the treatment of different human autoimmune disorders, including T1DM (Marek-Trzonkowska et al. 2012; Phillips et al. 2017; Stojanovic et al. 2017). Nonetheless, the isolation, differentiation and expansion of such immune cells are tedious and raise concerns on the feasibility of this approach for therapeutics. Alternatively, mesenchymal stromal/stem cells (MSC) have gained increasing interest due to their immunomodulatory and regenerative properties as well as ease of isolation/purification from various adult tissues (Franquesa et al. 2012; Shen et al. 2021). As of 2022, approximately 1500 clinical trials using MSC were registered at www.clinical trials.gov for indications ranging from neurological disease to diabetes. Of particular appeal are umbilical cord MSC (UC-MSC) that, in addition to possess immunosuppressive and anti-inflammatory properties, also display low immunogenicity offering the prospect of allogeneic transplantation (Can et al. 2017). To date, several clinical trials using UC-MSC infusion into newly onset T1DM patients have been yet completed but generated conflicting outcomes. On one hand, two independent studies reported no improvement in beta cell mass or C-peptide preservation whereas Tregs were increased in one study but not in the other subsequent to a 1 and 2 year follow-up of a single UC-MSC infusion (Haller et al. 2011; Giannopoulou et al. 2014). On the other hand, two other studies claimed that both C-peptide and HbA1c levels were improved after UC-MSC infusion resulting in reduced daily insulin dosage for patients at more than 1 year post-infusion (Hu et al. 2013; Lu et al. 2021). A common denominator to all studies was the absence of adverse side effects related to infusion (Haller et al. 2011; Hu et al. 2013; Giannopoulou et al. 2014; Lu et al. 2021).

Given the high yield of healthy MSC that can be harvested from the umbilical cord combined with their immunomodulatory and regenerative properties, we sought to dissect the cellular and molecular events derived from UC-MSC intraperitoneal (i.p.) transplantation in the RIP-B7.1 mouse model of experimental autoimmune diabetes (EAD) in order to resolve pending discrepancies hindering further clinical studies. Of special relevance, transgene expression of the co-stimulatory B7.1 molecule in β-cells has shown to represent a highly reproducible EAD induction model (Rajasalu et al. 2004; Rajasalu et al. 2010; Cobo-Vuilleumier et al. 2018), by inducing a rapid and consistent insulitis mimicking closely the aggressive immune attack occurring in younger children with type 1 diabetes (Craig et al. 2019).

Overall, we show that a single i. p. injection of UC-MSC could delay the onset of insulitis and hyperglycemia in RIP-B7.1 mice. Mechanistically, we show that UC-MSC i. p. transplantation drastically modified the composition and phenotype of peritoneal fluid cells, by mobilizing myeloid-derived suppressor cells (MDSC), T Cells and regulatory T Cells (Tregs). Interestingly, similar regulatory cells responses were further detected in diverse degrees into the spleen and pancreatic lymph nodes, in a timely orchestrated process correlating ultimately with a significantly lower pancreatic infiltration of T and B Cells as well as pro-inflammatory macrophages.

## Results

### Phenotypic characterization of umbilical cord mesenchymal stromal cells

Consistent with mesenchymal stromal/stem cells (MSC) characteristics, umbilical cord MSC (UC-MSC) derived from E17 pregnant FVB mice displayed the typical fibroblastic morphology of MSC and accordingly expressed pericytes (α-SMA, desmin, PDGR-β and NG2) and mouse MSC (CD29, Sca-1 and CD44) markers (Supplementary Figures S1A–C). Consistent with their stemness properties, UC-MSC were also shown to possess mesodermal differentiation capacity as evidenced by their ability to acquire adipocytes, chondrocytes and osteocytes characteristics upon culture into specific lineage inductive media (Supplementary Figures S1D–F).

### Intraperitoneal UC-MSC transplantation delays EAD onset, reduces insulitis and normalizes the plasmatic cytokines profile in immunized-RIP-B7.1 mice

**FIGURE 1:**
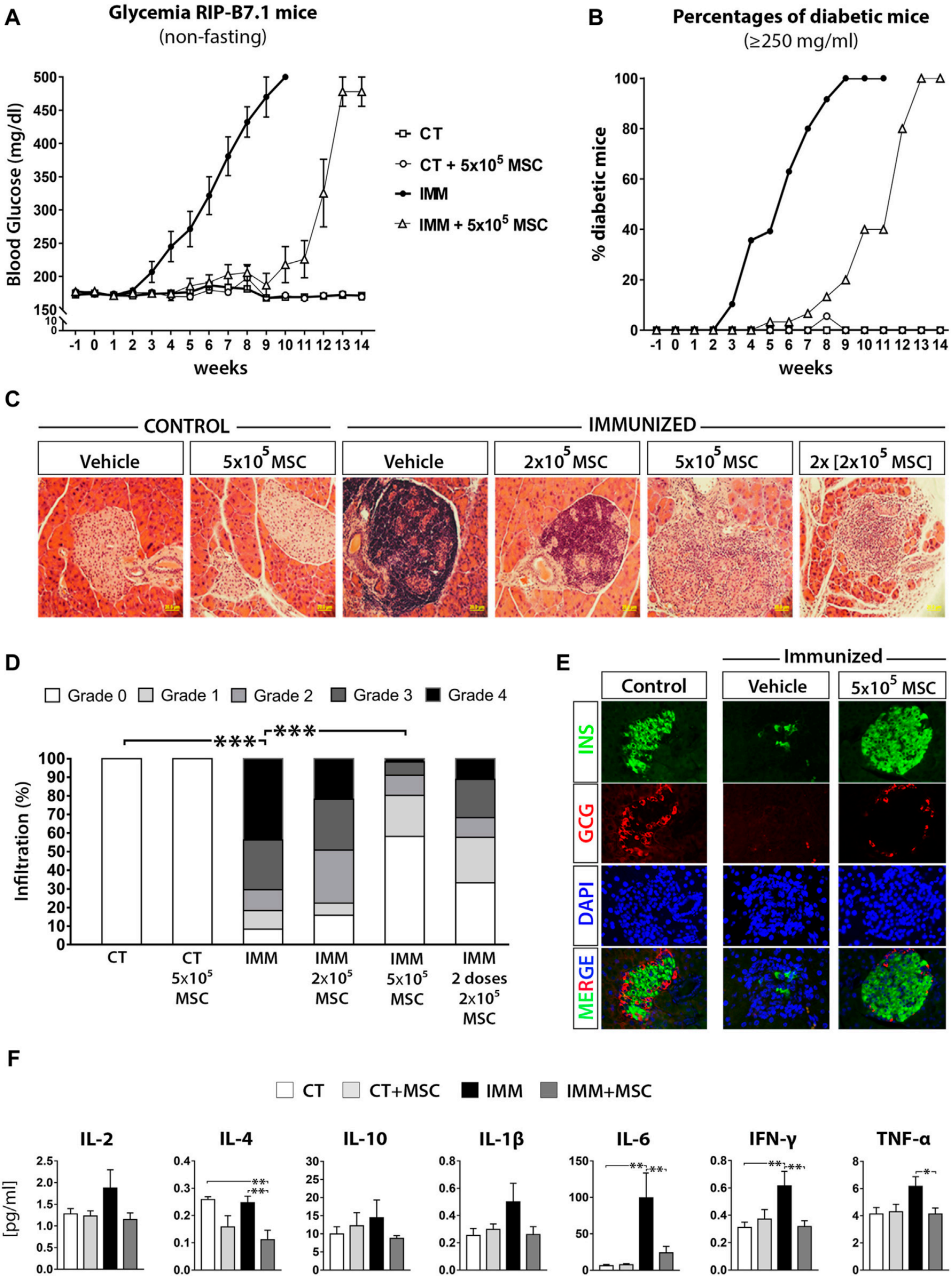
UC-MSC transplantation delay the onset of experimental autoimmune diabetes (EAD) in immunized RIP-B7.1 mice, reducing insulitis and normalizing plasmatic cytokine profile during the protective phase. **(A)** Summary measurements of non-fasting blood glucose in control (CT) and immunized (IMM) RIP-B7.1 mice transplanted with vehicle or 5 × × 10^5^ UC-MSC, recorded until 14 weeks post transplantation. **(B)** Summary percentages of diabetic RIP-B7.1 mice (≥250 mg/dL) into different experimental groups. **(A, B)** Values are means ± s. e.m. of *n* = 18 mice for CT and CT+5 × 10^5^ UC-MSC, *n* = 28 for IMN and *n* = 31 for IMM+5 × 10^5^ UC-MSC. **(C)** Representative hematoxylin and eosin (H&E) histological staining of pancreatic sections from CT and IMM-RIP-B7.1 mice at 7 weeks post-transplantation with vehicle or 5 × 10^5^ UC-MSC (40X magnification). **(D)** Insulitis scores as a grade of 0–4 according to percentage of infiltrated islets. (*n* = 4 mice per group). **(E)** Pancreatic sections from CT and IMM RIP-B7.1 mice at 7 weeks post-transplantation with vehicle or 5 × 10^5^ UC-MSC were co-immunostained for insulin (green) and glucagon (red). Nuclei are stained with DAPI (blue). Representative single-channel fluorescence images are shown individually and merged. 40X magnification. **(F)** Plasmatic cytokines profile from CT and IMM RIP-B7.1 mice at 7 weeks after vehicle or 5 × 10^5^ UC-MSC transplantation. Values are mean ± SE cytokine concentration [pg/mL]. CT, *n* = 8; CT + UC-MSC, *n* = 8; IMM, n = 10; IMM + UC-MSC, n = 10. **(D, F)** *, *p* ≤ 0.05; **, *p* ≤ 0.03; ***, *p* ≤ 0.01, one-way ANOVA.

Increasing amounts of UC-MSC were intraperitoneally (i.p.) transplanted into either immunized (IMM) or not (CT) RIP-B7.1 mice and glycemia was monitored up to 8-weeks post treatment (Supplementary Figure S2). A single i. p. dose of 5 × 10^5^ UC-MSC provided the most effective reduction of hyperglycemia as compared to either a single or two doses of 2 × 10^5^ UC-MSC (Supplementary Figure S2A). Long-term follow-up experiments indicated that a single i. p. dose of 5 × 10^5^ UC-MSC provided a delay of approximately 7 weeks in the EAD onset of IMM-RIP-B7.1 mice, after when 100% of them became hyperglycemic at 13-14 weeks post-transplantation (Figures 1A, B). Control RIPB7.1 mice (not immunized) which were transplanted with 5 × 10^5^ UC-MSC didn´t show any obvious alterations in terms of overall health status, glycemia (Figure 1A) or weight (data not shown) by comparison to their non-transplanted counterparts.

To understand how UC-MSC transplantation protected temporally IMM-RIP-B7.1 mice against the programmed EAD, we first analyzed the presence of immune cells infiltration within islets (insulitis) at 7 days and 7 weeks post-transplantation (Figure 1C). A single dose of 5 × 10^5^ UC-MSC produced the best protection against the development of the highest grades of insulitis in IMM-RIP-B7.1 mice (Figure 1D), and preserved islets integrity and normal α- and β-cell content (Figure 1E). Correlating with these results, IMM-RIP-B7.1 mice transplanted with UC-MSC had reduced plasmatic concentrations of the pro-inflammatory cytokines IL-6, IFN-γ and TNF-α as compared to vehicle-treated IMM-RIP-B7.1 mice (Figure 1F).

### Biodistribution of transplanted UC-MSC

**FIGURE 2:**
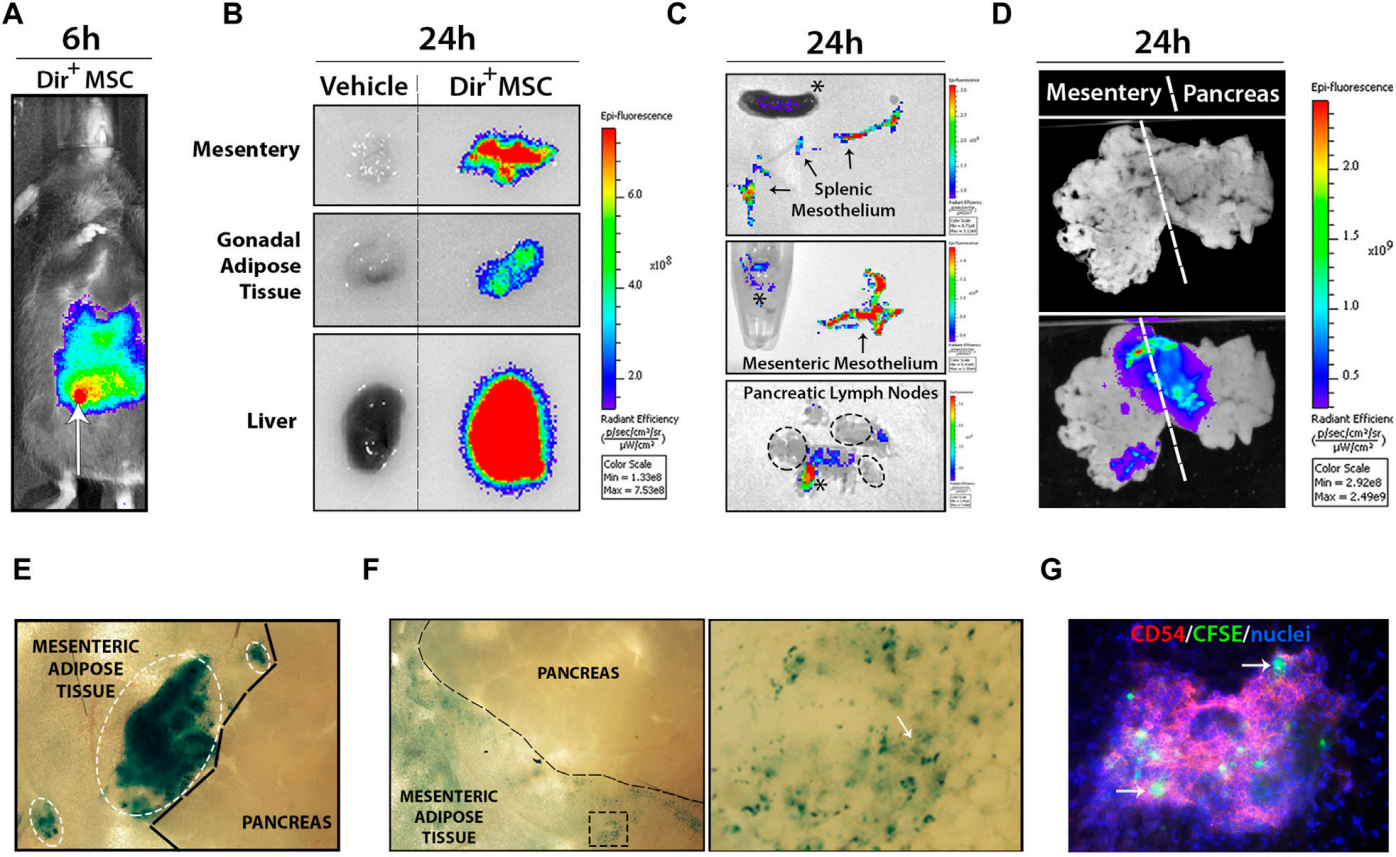
Transplanted UC-MSC rapidly spread inside the peritoneal cavity and adhere to the mesothelium surface of several peritoneal organs. **(A)** Shows wide dissemination of 5 × 10^5^ Dir^+^ UC-MSC through the peritoneal cavity at 6 h post-transplantation (arrow points to the injection site). **(B)** Shows comparative Dir^+^ fluorescence levels into mesentery, gonadal adipose tissue and liver section from vehicle or Dir^+^ UC-MSC transplanted IMM mice, at 24 h post-transplantation. **(C)** Upper and middle images show comparative Dir^+^ fluorescence into the separated splenic and mesenteric mesothelium (arrows), respectively. Lower image shows Dir^+^ fluorescence signal in pancreatic lymph nodes (PLN), which was externally restricted to peripheral adipose tissue (asterisks), but not to PLN (ellipse). **(D)** Whole-mount visualization of connected mesentery and pancreas at 24 h post-transplantation of Dir^+^ UC-MSC and after washing in PBS allows the visualization of small to large Dir^+^ UC-MSC aggregates adhered on their surface. **(E)** *En face* bright field picture of mesentery and pancreas surface at 24 h post-transplantation of Dir^+^ UC-MSC, showing small to large blue Dir^+^ UC-MSC aggregates (ellipses) adhered on top of the mesenteric adipose tissue mesothelium but not on top of the pancreatic mesothelium. **(F)** *En face* bright field picture of mesentery and pancreas surface at 7 days post-transplantation of Dir^+^ UC-MSC. At that time post-transplantation, Dir^+^ aggregates were not observable on top of the mesentery surface (left image). By contrast, many areas of the mesenteric adipose tissue displayed residual Dir^+^ dots in surface cells (enlarged spot). **(G)** *En face* immunofluorescence picture of the mesentery surface showing Dir^+^ cells inside a milky spot (depicted by CD54-PE^high^ lymphocytes) which should likely correspond to macrophages with internalized Dir^+^ residues.

*In vivo* imaging system (IVIS) was used to track Dir^+^-labelled UC-MSC after i. p. transplantation (Figures 2A, B). Dir^+^ signals rapidly disseminated through the peritoneal cavity and incorporated to the different visceral organs tested, including the mesentery, gonadal fat depots and liver at 24 h post-transplantation. Strong Dir^+^ signals detected in the stroma of fresh liver section clearly indicate an accumulation of phagocytized Dir^+^ UC-MSC-derived residues (Figure 2B). Analysis of the spleen and mesentery suggested that Dir^+^ UC-MSC had not migrated massively into their stroma since the majority of Dir^+^ signal was concentrated into their outermost mesothelium (Figure 2C). In line with this finding, pancreatic lymph nodes (PLN) were also found to lack detectable internal Dir^+^ signal, which was instead detected onto adipose tissue surrounding PLN (Figure 2C). Finally, some large Dir^+^ UC-MSC aggregates were found adhered onto the mesenteric mesothelium in close proximity to the pancreas in most UC-MSC transplanted RIP-B7.1 mice, which supports a local action of UC-MSC to the target pancreas (Figures 2D, E). However, most of the Dir^+^ UC-MSC aggregates initially detected as adhered onto the mesenteric surface at day 1 post-transplantation were by contrast undetectable after 7 days when only traces of Dir^+^ signals inside mesothelium cells which could correspond to macrophages (Figures 2E, F). Finally, at 7 days post-transplantation of CFSE^+^ UC-MSC, fluorescent cells consistent with UC-MSC or phagocytes bearing UC-MSC-derived CFSE^+^ residues were detected inside CD54^hi^ milky spots in the mesenteric submesothelium (Figure 2G).

### UC-MSC are massively targeted by peritoneal macrophages

**FIGURE 3:**
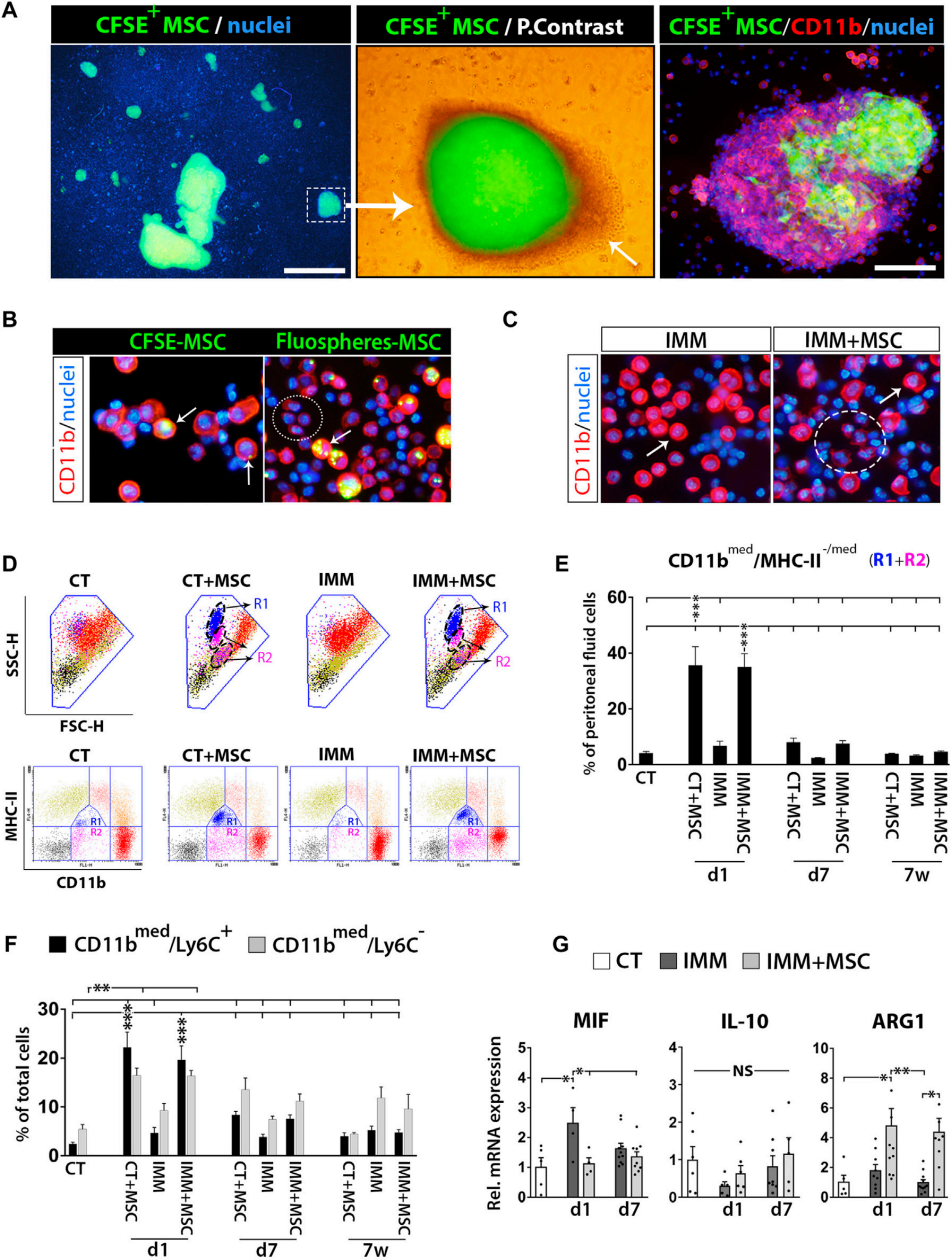
Intraperitoneally transplanted UC-MSC are targeted by peritoneal macrophages in a process accompanied by a strong mobilization of neutrophils and myeloid-derived suppressor cells in the peritoneal fluid. **(A)** Left image, shows low 2X magnification fluorescence picture of a peritoneal drainage collected at 24 h post-transplantation of CFSE^+^ UC-MSC and showing presence of many small to very large CFSE^+^ UC-MSC clusters. Scale bar is 500 µm. Middle image, merged fluorescent and light photograph of CFSE^+^ UC-MSC aggregates showing a core of CFSE^+^ UC-MSC surrounded by several layers of CSFE^neg^ external cells (arrow). Right image, CD11b-PE staining of peritoneal lavage cells helped to visualize how CFSE^+^ UC-MSC aggregates had an external layer of CD11b^high^/CSFE^neg^ peritoneal macrophages. Scale bar is 100 µm. **(B)** Shows CD11b-PE stained peritoneal fluid cells (PFC) collected by from IMM-RIP-B7.1 mice at 24 h post-transplantation of CSFE^+^ UC-MSC, or UC-MSC bearing phagocytosed Fluospheres. Note how, UC-MSC-derived fluorescent residues are by majority internalized into large CD11b^high^ peritoneal macrophages (arrows) but not into small CD11b^low^ expressing myeloid cells (ellipse). **(C)** CD11b-PE staining of PFC collected at 24 h from immunized mice transplanted with vehicle (IMM) or UC-MSC (IMM + MSC). Note the presence of numerous CD11b^low^ expressing myeloid cells (ellipse) among PFC from IMM + MSC mice. Their smaller size is distinguishable from CD11b^high^ large peritoneal macrophages (arrow). **(D, E)** Flow cytometric quantification of CD11b^med^/MHC-II^-/med^ myeloid-derived suppressor cells within PFC. **(D)** CD11b and MHC-II (I/A-I/E) expression analysis onto PFC from CT or IMM RIP-B7.1 mice 24 h post-transplantation. UC-MSC transplantation induced mobilization of two myeloid subpopulations with distinct SSC-H/FSC-H characteristics and MHC-II/CD11b costaining patterns. Upper, SSC-H/FSC-H dot plots of gated PFC (blue line area). Note how cells display distinct coloration according to their CD11b and MCH-II expression patterns clustered into distinct regions (lower). PFC from CT + MSC and IMM + MSC mice contain display increased CD11b^med^/MHC-II^med^ (R1) and CD11b^med^/MHC-II^neg-low^ (R2) myeloid cells subsets, which are delimited by a dashed ellipse into SSC-H/FSC-H dot plots. **(E)** Quantification of CD11b^med^/MHC-II^-/med^ myeloid cells (R1+R2) percentages. **(F)** Quantification of CD11b^med^/Ly6C^−^ myeloid cells and CD11b^med^/Ly6C^+^ myeloid-derived suppressor cells (MDSC) percentages. **(E, F)** Analysis performed onto PFC from CT and IMM RIP-B7.1 mice transplanted with vehicle or 5 × 10^5^ UC-MSC, at time of 1 day, 7 days and 7 weeks post-transplantation. **(G)** Q-PCR analysis of macrophage inhibitory factor (MIF), interleukin-10 (IL-10) and arginase I (ARG1) mRNA expression in PFC collected at 1 and 7 days after UC-MSC transplantation. **(E–G)** Values are mean ± s. e.m of n ≥ 5 mice for each experimental group. Results show relative mRNA expression to CT mice (value set as 1). *, *p* ≤ 0.05; **, *p* ≤ 0.03; ***, *p* ≤ 0.01, one-way ANOVA. NS is for not significant differences.

To start deciphering the immune protection mechanisms triggered by UC-MSC i. p. transplantation, we first analyzed peritoneal fluid cells (PFC) from IMM mice transplanted or not with fluorescently labelled UC-MSC (Supplementary Figure S3). Peritoneal drainages collected at 24 h post-transplantation with CFSE^+^ UC-MSC helped the visualization of numerous small to large cellular aggregates, consisting of a core of CFSE^+^/CD11b^neg^ UC-MSC surrounded by several layers of CFSE^−^/CD11b^hi^ myeloid cells (Figure 3A). Examination of PFC at day 1 post-transplantation of fluorescently-labelled UC-MSC, indicated that part of CD11b(hi) large peritoneal macrophages presented fluorescent residues in their cytoplasm, indicating they are actively involed in UC-MSC phagocytosis (Figure 3B). Most UC-MSC/myeloid cells aggregates were collected as free-floating aggregates within peritoneal lavages, while by contrast only a minor portion of them were found firmly attached on top of mesenteric adipose depots (Figures 2D, E).

### UC-MSC transplantation triggers a rapid and massive recruitment of myeloid-derived suppressor cells (MDSC) in the peritoneal fluid

Analysis of PFC collected from RIP-B7.1 mice at day 1 post-transplantation evidenced how many of them corresponded to small CD11b^med^ myeloid cells (Figure 3C), clearly distinguishable from large rounded CD11b^hi^ peritoneal macrophages (Cassado Ados et al. 2015). Correlating with this finding, PFC from both CT- and IMM-RIP-B7.1 mice transplanted with UC-MSC were found to contain approximately 30% of CD11b^med^ myeloid cells, consisting mostly of [CD11b^med^/MHC-II^low^] cells and in lower extent of [CD11b^med^/MHC-II^neg^] cells (Figures 3D, E). Further analysis indicated that these infiltrated CD11b^med^ myeloid cells were also composed by both CD11b^med^/Ly6C^+^ and CD11b^med^/Ly6C^−^ myeloid cells (Figure 3F), being Ly6C expressed onto mouse MDSC (Saiwai et al. 2013). A more detailed analysis of these Ly6C^+^ MDSC indicated that they corresponded to granulocytic [CD11b^med^/LY6C^+^/SCC-H^hi^] and monocytic [CD11b^med^/LY6C^hi^/SCC-H^med^] MDSC (data not shown).

Quantitative real-time PCR (qPCR) analyses revealed that PFC from transplanted IMM-RIP-B7.1 mice had significantly higher levels of the enzyme arginase I (ARG1) as compared to either CT or IMM mice (Figure 3G), which is highly expressed in MDSC (Gabrilovich and Nagaraj 2009) and acts as a major immunosuppression mechanism of T Cells responses through depletion of L-arginine (Bronte and Zanovello 2005; Munder 2009). Conversely, the proinflammatory cytokine macrophage inhibitory factor (MIF) which expression was upregulated in PFC from IMM-RIP-B7.1 mice was by contrast downregulated in IMM-RIP-B7.1 mice transplanted with UC-MSC (Figure 3G).

### Initial acute peritoneal immune response induced by UC-MSC transplantation is followed by a massive activation of peritoneal macrophages and mobilization of T cells in the peritoneal fluid

**FIGURE 4:**
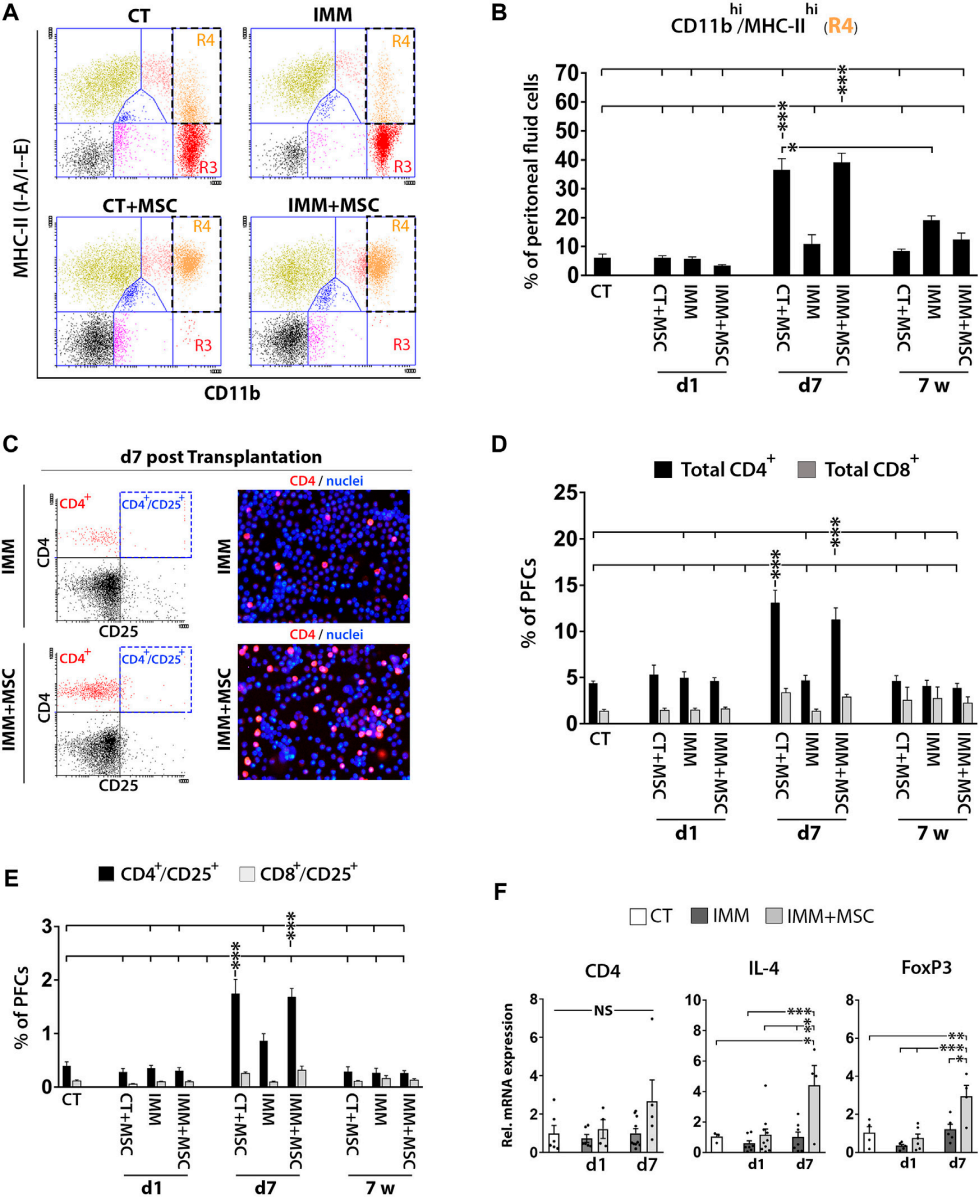
UC-MSC transplantation induces in a second step a strong activation of peritoneal macrophages and mobilization of T Cells and regulatory T Cells. **(A, B)** Peritoneal macrophages are massively activated at 7 days post-transplantation, but return to a lower activation state after 7 weeks post-transplantation. **(A)** Representative CD11b and MHC-II expression dot plots of PFC from 7 days transplanted CT or IMM RIP-B7.1 mice with vehicle or 5 × 10^5^ UC-MSC. Note how CD11b^hi^/MHC-II^neg-low^ peritoneal macrophages (R3 region) in CT and IMM mice shifted to an activated CD11b^hi^/MHC-II^hi^ phenotype (R4 region) in CT + MSC and IMM + MSC mice. **(B)** Quantification of CD11b^hi^/MHC-II^hi^ activated macrophages percentages within PFC. **(C–F)** Peritoneal fluid contains increased T Cells and activated T Cells percentages and regulatory T Cells markers at 7 days post-transplantation. **(C)** Representative CD4 and CD25 expression dot plots and CD4-PE stainings of PFC from IMM and IMM + MSC mice at 7 days post-transplantation. **(D)** Quantification of total CD4^+^ and CD8^+^ T Cells percentages within PFC. **(E)** Flow cytometric quantification of CD4^+^/CD25^+^ and CD8^+^/CD25^+^ activated T Cells percentages within PFC. **(B, D, E)** PFC were collected from CT and IMM RIP-B7.1 mice, at 24 h, 7 days and 7 weeks post-transplantation with vehicle or 5 × 10^5^ UC-MSC. **(F)** Q-PCR analysis of CD4, interleukin-4 (IL-4) and forkhead box P3 (FoxP3) mRNA expression in PFC collected from CT and immunized RIP-B7.1 mice transplanted with vehicle (IMM) or 5 × 10^5^ UC-MSC (IMM + MSC) at day 1 and 7 post-transplantation. Results show relative mRNA expression to CT mice (value set as 1). **(B, D, E F)** Values are mean ± s. e.m of n ≥ 5 mice for each experimental group. *, *p* ≤ 0.05; **, *p* ≤ 0.03; ***, *p* ≤ 0.01, one-way ANOVA. NS is for not significant differences.

Analysis of PFC collected from the different experimental groups could evidence how resident peritoneal macrophages initially detected as CD11b^hi^/MHC-II^low^ cells at day 1 post-transplantation had further shifted their immunophenotype to activated CD11b^hi^/MHC-II^high^ macrophages at 7 days post-transplantation (Figures 4A, B). Relevantly, PFC collected in mice at 7 days post-transplantation were also found to contain higher percentages of CD4^+^ and CD8^+^ T Cells (Figures 4C, D), suggesting that an active interaction between peritoneal macrophages and T Cells was possibly occurring at that time post-transplantation. Interestingly, percentages of activated CD4^+^/CD25^+^ and CD8^+^/CD25^+^ T Cells were also increased at 7 days post-transplantation (Figure 4E) and correlated with a higher expression levels by PFC of transcripts encoding interleukin-4 (IL-4) and FoxP3 (Figure 4F), which could indicate that part of the increase in activated CD4^+^/CD25^+^ T Cells correspond to regulatory T Cells (Tregs).

### Spleens of UC-MSC transplanted mice display increased MDSC percentages and anti-inflammatory/immunosuppressive markers

**FIGURE 5:**
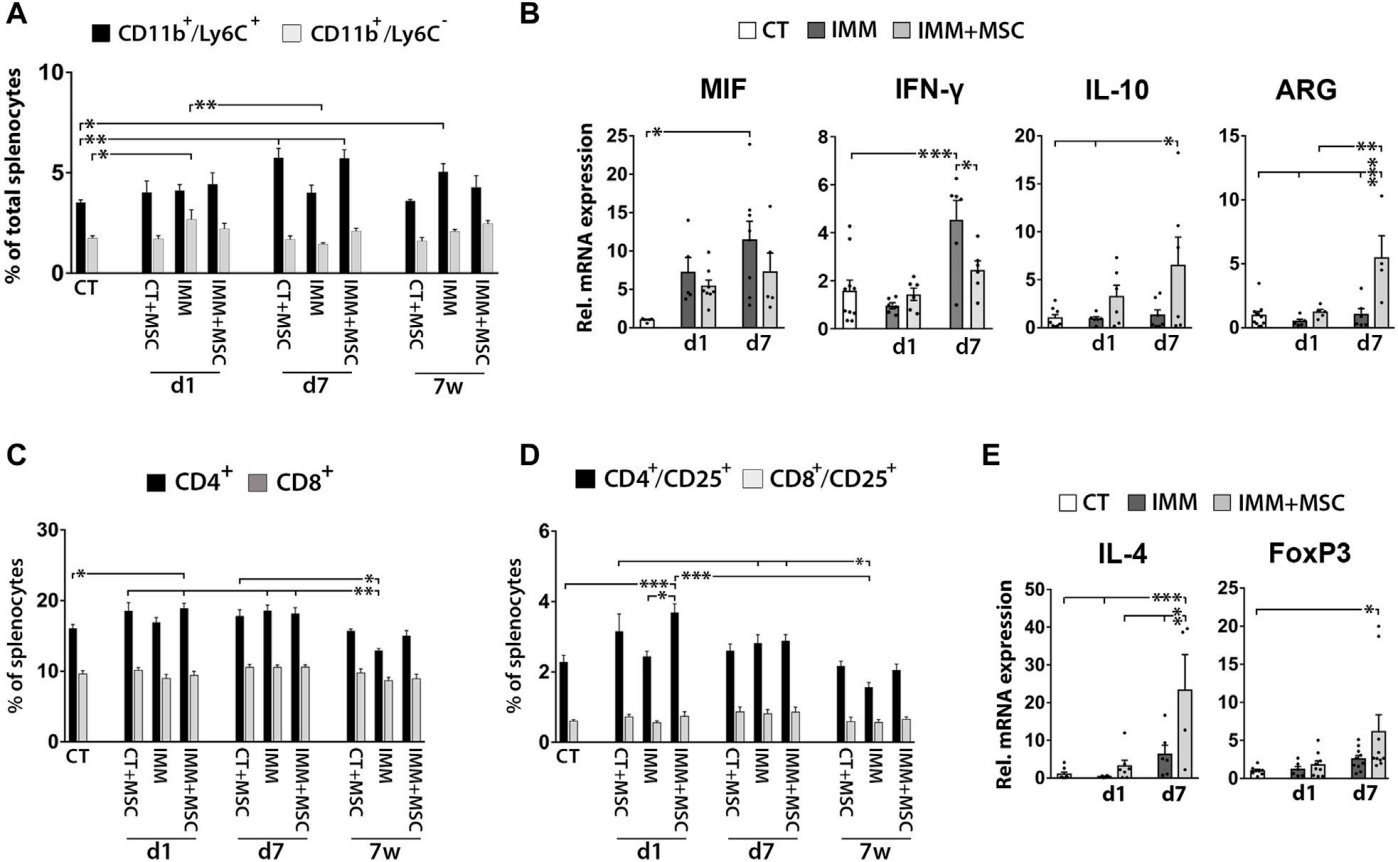
UC-MSC transplantation induces splenic anti-inflammatory and immunosuppressive responses in immunized RIP-B7.1 mice. **(A)** Quantification of splenic CD11b^+^/Ly6C^−^ myeloid cells and CD11b^+^/Ly6C^+^ myeloid-derived suppressor cells (MDSC). Note how both CT and IMM mice at 7 days post-transplantation display increased splenic percentages of CD11b^+^/Ly6C^+^ MDSC. **(B)** Q-PCR quantification of MIF, IL-10 and ARG1 mRNA expression. **(C–E)** Splenocytes from UC-MSC transplanted IMM mice display increased percentages of total and activated CD4^+^ T Cells and higher expression of the associated regulatory T Cells markers IL-4 and forkhead box P3 (FoxP3), compared to CT mice. **(C)** Quantification of total splenic CD4^+^ and CD8^+^ T Cells percentages. **(D)** Quantification of total splenic activated CD4^+^/CD25^+^ and CD8^+^/CD25^+^ T Cells percentages. **(A, C, D)** Splenocytes were collected from CT and IMM RIP-B7.1 mice, at 24 h, 7 days and 7 weeks post-transplantation with vehicle or 5 × 10^5^ UC-MSC. **(E)** Q-PCR quantification of interleukin-4 (IL-4) and FoxP3 mRNA expression. **(B, E)** Splenocytes collected from CT and immunized RIP-B7.1 mice transplanted with vehicle (IMM) or 5 × 10^5^ UC-MSC (IMM + MSC) at day 1 and 7 post-transplantation. Results show relative mRNA expression to CT mice (value set as 1). **(A–E)** Values are mean ± s. e.m of n ≥ 5 mice for each experimental group. *, *p* ≤ 0.05; **, *p* ≤ 0.03; ***, *p* ≤ 0.01, one-way ANOVA. NS is for not significant differences.

As previously observed in the peritoneal fluid, mice analyzed at 7 days post-transplantation also displayed increased percentages of CD11b^+^/Ly6C^+^ MDSC in their spleen (Figure 5A), consisting of granulocytic [CD11b^+^/Ly6C^low/med^/SSC-H^high^] and monocytic [CD11b^+^/Ly6C^hi^/SSC-H^med^] MDSC (Supplementary Figure S3). Correlating with these findings, splenocytes from UC-MSC transplanted IMM-RIP-B7.1 mice also up-expressed interleukin-10 (IL-10) and ARG1, two genes which are highly expressed by MDCS (Figure 5B). Relevantly, and as similarly observed in PFC, splenocytes from IMM-RIP-B7.1 mice also displayed significantly higher expression of MIF as compared to CT mice (Figure 5B), confirming thus that MIF up-expression likely plays a relevant role in the EAD of IMM-RIP-B7.1 mice. However, and by contrast to results initially observed in PFC (Figure 3G), no significant differences for MIF expression were observed in response to UC-MSC transplantation in IMM-RIP-B7.1 mice, even if the results at days 1 and 7 post-transplantation show a pattern indicating a possible reduction in its expression (Fig. 5 b).

### UC-MSC transplantation transiently increases activated CD4^+^/CD25^+^ T cells and tregs markers in the spleen of immunized-RIP-B7.1 mice

We next assessed whether UC-MSC transplantation alters splenic T Cells percentages (Figures 5C, D). Although no drastic changes were observed over time post-transplantation, UC-MSC transplanted IMM-RIP-B7.1 mice displayed a transient increase in total CD4^+^ T Cells and CD4^+^/CD25^+^ activated T Cells at day 1 post-transplantation (Figures 5C, D). Accordingly, splenocytes of UC-MSC transplanted IMM-RIP-B7.1 mice had higher transcript levels of the Treg markers IL-4 and FoxP3 (Figure 5E).

### UC-MSC transplantation moderately increases MDSC and activated T cells in pancreatic lymph nodes

**FIGURE 6:**
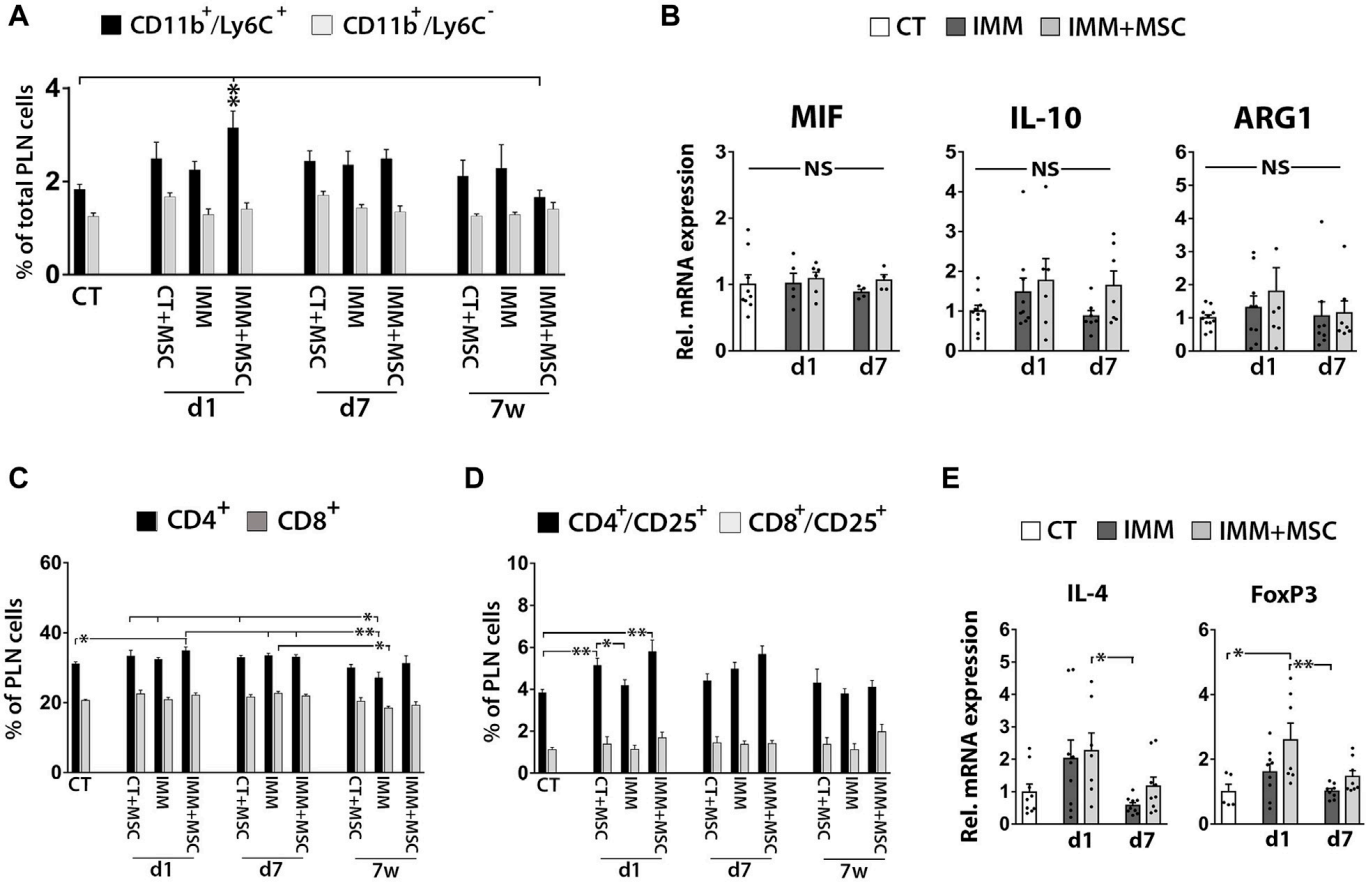
Immunized RIP-B7.1 transplanted with UC-MSC display transiently increased MDSC, activated T Cells and regulatory T Cells markers in pancreatic lymph nodes. **(A)** Quantification of CD11b^+^/Ly6C^−^ and CD11b^+^/Ly6C^+^ MDSC within pancreatic lymph nodes (PLN). Note how PLN from day 1-transplanted IMM mice display increased percentage of CD11b^+^/Ly6C^+^ MDSC. **(B)** Q-PCR analysis of MIF, IL-10 and ARG1 mRNA expression in PLN. Results show relative mRNA expression to CT mice (value set as 1). **(C–E)** PLN from day 1 UC-MSC transplanted IMM mice transiently display higher percentages of total and activated CD4^+^ T Cells, correlating with higher expression of the regulatory T Cells markers forkhead box P3 (FoxP3). **(C)** Summary flow cytometric quantification of total CD4^+^ and CD8^+^ T Cells in PLN. **(D)** Quantification of activated CD4^+^/CD25^+^ and CD8^+^/CD25^+^ T Cells in PLN. **(A, C, D)** Splenocytes were collected from CT and IMM RIP-B7.1 mice, at 24 h, 7 days and 7 weeks post-transplantation with vehicle or 5 × 10^5^ UC-MSC. **(E)** Q-PCR analysis of interleukin-4 (IL-4) and FoxP3 mRNA expression in PLN. **(B, E)** PLN were collected from CT and immunized RIP-B7.1 mice transplanted with vehicle (IMM) or 5 × 10^5^ UC-MSC (IMM + MSC) at day 1 and 7 post-transplantation. **(A–D)** Values are mean ± s. e.m of n ≥ 5 mice for each experimental group. *, *p* ≤ 0.05; **, *p* ≤ 0.03; ***, *p* ≤ 0.01, one-way ANOVA. NS is for not significant differences.

Although FC analysis indicated a transient increase of CD11b^+^/Ly6C^+^ MDSC in pancreatic lymph nodes (PLN) from IMM-RIP-B7.1 mice at day 1 post-transplantation (Figure 6A), no obvious differences for MIF, IL-10 or ARG1 mRNA expression were observed between experimental groups (Figure 6B). Furthermore, and as previously observed in splenocytes, PLN cells from day 1 transplanted IMM-RIP-B7.1 mice also displayed higher percentages of both CD4^+^ T Cells and CD4^+^/CD25^+^ activated T Cells when compared to CT mice (Figures 6C, D), but however did not show significant increases in IL-4 and Foxp3 mRNA expression compared to non-transplanted IMM-RIP-B7.1 mice (Figure 6E).

### UC-MSC transplantation induces a transient increase of activated B cells in the spleen and pancreatic lymph nodes of immunized RIP-B7.1 mice

Flow cytometry analysis revealed higher percentages of activated CD19^+^/CD25^+^ B Cells in the spleen and pancreatic lymph nodes of IMM-RIP-B7.1 mice, at 1 day after UC-MSC transplantation (Supplementary Figure S4F, G). Percentages of activated B Cells percentages among PFCs were in turn decreased in both CT- and IMM-RIP-B7.1 mice, at 7 days post-transplantation (Supplementary Figure S4E).

### UC-MSC transplantation reduces pancreatic infiltration of T and B cells and pro-inflammatory macrophages in immunized RIP-B7.1 mice

**FIGURE 7:**
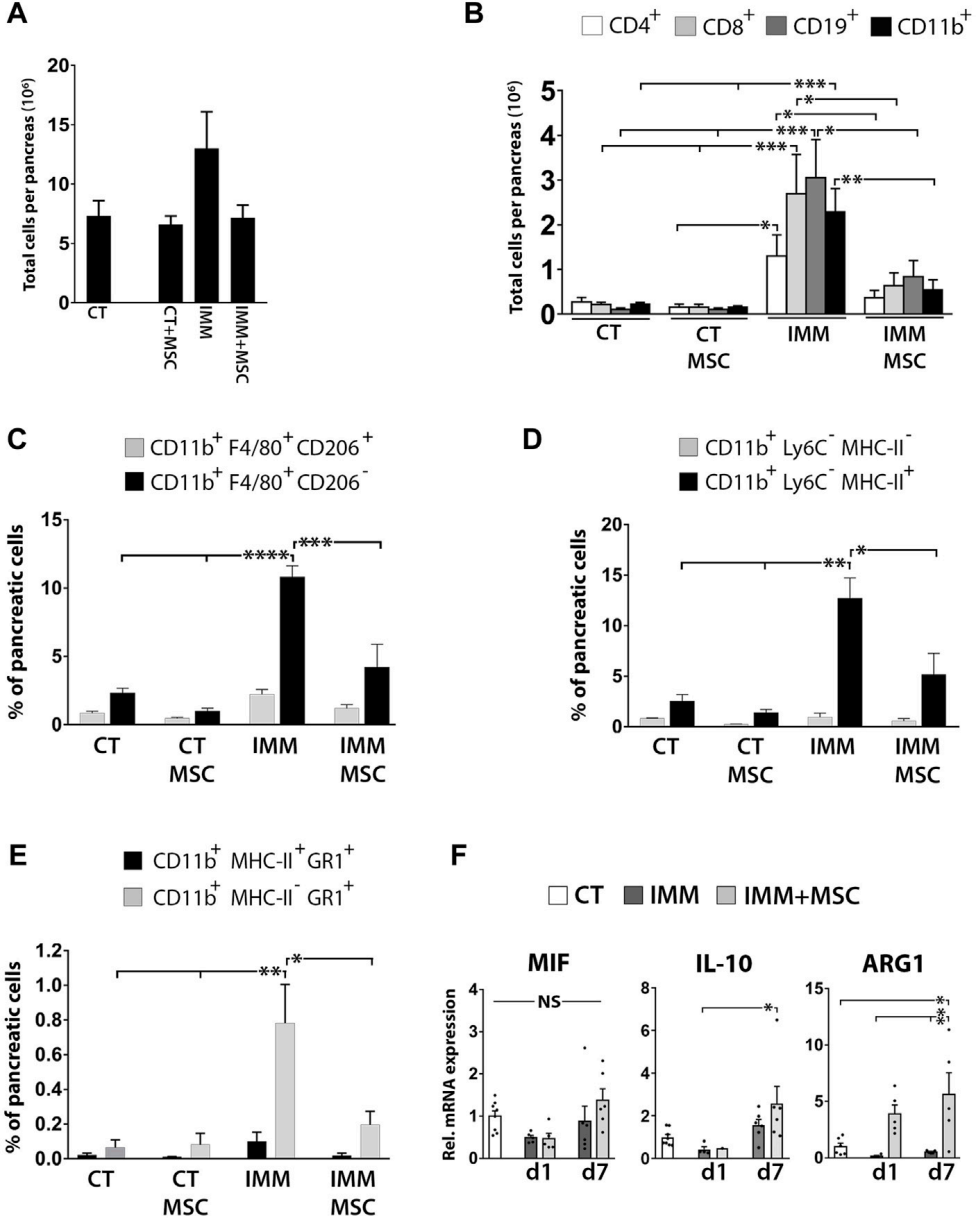
UC-MSC transplantation significantly reduces pancreatic leukocytes infiltration in IMM-RIP-B7.1 mice. **(A)** Total pancreatic stromal cells counts obtained from experimental groups at 7 weeks after UC-MSC transplantation. **(B)** Quantification of total numbers of CD4^+^ and CD8^+^ T Cells, CD19^+^ B Cells and CD11b^+^ myeloid cells per pancreas in CT or IMM RIP-B7.1 mice at 7 weeks post-transplantation with vehicle or 5 × 10^5^ UC-MSC. **(C)** Quantification of [CD11b^+^;F4/80^+^;CD206^-^] and [CD11b^+^;F4/80^+^;CD206^+^] pancreatic macrophages subsets. **(D)** Quantification of [CD11b^+^;Ly6C^−^;MHC-II^-^] and [CD11b^+^;Ly6C^−^;MHC-II^+^] pancreatic myeloid cells subsets. **(E)** Quantification of [CD11b^+^;MHC-II^+^;GR1^+^] and [CD11b^+^;MHC-II^-^;GR1^+^] pancreatic myeloid cells subsets. **(A–E)** Analysis performed onto pancreatic stromal cells were collected from CT- or IMM-RIP-B7.1 mice at 7 weeks post-transplantation with vehicle or 5 × 10^5^ UC-MSC. **(F)** Q-PCR analysis of MIF, IL-10 and ARG1 mRNA expression in pancreatic stromal cells from CT and immunized RIP-B7.1 mice transplanted with vehicle (IMM) or 5 × 10^5^ UC-MSC (IMM + MSC) at day 1 and 7 post-transplantation. Results show relative mRNA expression to CT mice (value set as 1). **(B–F)** Values are mean ± s. e.m of n ≥ 5 mice for each experimental group. *, *p* ≤ 0.05; **, *p* ≤ 0.03; ***, *p* ≤ 0.01, one-way ANOVA. NS is for not significant differences.

We next analyzed leukocytes infiltration in the pancreas at day 1 and 7 as well as at 7 weeks post-transplantation and found that as early as 15 days post-immunization (corresponding to 7 days post-transplantation), IMM-RIP-B7.1 mice already displayed a significant increase of both CD4^+^ T- and CD19^+^ B Cells among pancreatic stromal cells (Supplementary Figure S5). At 8 weeks post-immunization (7 weeks post-transplantation), IMM-RIP-B7.1 mice which already had developed severe insulitis and hyperglycemia (Figure 1) displayed the highest increase of CD11b^+^ myeloid cells, CD4^+^ and CD8^+^ T Cells and CD19^+^ B Cells (Supplementary Figure S5). Establishment of total cell counts per pancreas at that time post-transplantation (Figure 7A) could evidence how UC-MSC-transplanted IMM-RIP-B7.1 mice displayed significantly lower numbers of infiltrated CD4^+^ and CD8^+^ T Cells (*p < 0.05*), CD19^+^ B Cells (*p < 0.05*) and CD11b^+^ myeloid cells (*p ≤ 0.03*) as compared to vehicle-treated IMM-RIP-B7.1 mice (Figure 7B).

### UC-MSC transplantation significantly reduces pancreatic infiltration of pro-inflammatory macrophages and MDSC in hyperglycemic immunized-RIP-B7.1 mice

The immunophenotype of CD11b^+^ myeloid cells infiltrated in pancreases of hyperglycemic IMM-RIP-B7.1 mice was further characterized by analyzing co-expression of the myeloid-related markers F4/80, CD206, MHC-II, Ly6C and Gr1. At 8 weeks post-immunization (7 weeks post-transplantation), approximately 80% of infiltrated myeloid cells into pancreases of IMM-RIP-B7.1 mice were identified as [CD11b^+^/F4/80^+^/CD206^-^] cells (Figure 7C), which are consistent with M1 pro-inflammatory macrophages (Antonios et al. 2013; Cassado Ados et al. 2015).

Additionally, and in a similar way, near 90% of pancreatic infiltrated myeloid cells corresponded to [CD11b^+^/Ly6C^−^/MHC-II^+^] cells (Figure 7D), an immunophenotype which is mostly consistent with activated macrophages (Gordon and Taylor 2005), but that also identifies dendritic cells subsets (Welzen-Coppens et al. 2012). Interestingly, islet-resident macrophages in NOD mice were also shown to be identified as MHC-II^hi^ cells (Ferris et al. 2017). In contrast, UC-MSC-transplanted IMM-RIP-B7.1 mice displayed a significantly reduced percentages of both [CD11b^+^/F4/80^+^/CD206^-^] and [CD11b^+^/Ly6C^−^/MHC-II^+^] inflammatory macrophages, which is in agreement with their reduced hyperglycemia as compared to non-transplanted IMM mice (Figures 7B, D). Of particular interest is also our finding that nearly 10% of myeloid cells infiltrated into pancreases of IMM-RIP-B7.1 mice were characterized as [CD11b^+^/MHC-II^neg/low^/Gr1^+^] cells (Figure 7E) an immunophenotype which is consistent with MDSC (Gabrilovich and Nagaraj 2009). Interestingly, our results indicated that percentages of [CD11b^+^/MHC-II^neg/low^/Gr1^+^] MDSC were also significantly reduced in UC-MSC transplanted IMM-RIP-B7.1 mice to levels closer to those of RIP-B7.1 control mice (Figure 7E).

However, and in contrast with these results, pancreatic stromal cells from IMM-RIP-B7.1 mice did not express higher levels of the immunosuppressive enzyme ARG1 which was instead expressed at higher levels in UC-MSC transplanted IMM-RIP-B7.1 mice (Figure 7F).

## Discussion

Long-term follow-up experiments indicated that a single i. p. transplantation of 5 × 10^5^ UC-MSC in immunized RIP-B7.1 mice could efficiently delay the onset of EAD through induction of multiple anti-inflammatory and immunosuppressive responses into the peritoneal cavity, spleen and pancreatic lymph nodes, which ultimately correlated with lower plasmatic proinflammatory cytokines concentrations and a reduced pancreatic leukocytes infiltration.

The i. p. cells transplantation approach has gained increasing interest in recent years, principally as local approach to treat peritoneal fibrosis or inflammatory or/and autoimmune diseases affecting visceral organs (Bertram et al. 1999; Li et al. 2009; Wakabayashi et al. 2014; El-Hossary et al. 2016).

Interestingly, a study reported the successful attenuation of streptozotocin-induced diabetes *via* systemic transplantation of human UC-MSC, while in contrast no effectiveness was obtained through the i. p. transplantation route (El-Hossary et al. 2016). Contrary to these outcomes, our results suggest that the i. p. transplantation of heterologous UC-MSC is an effective transplantation approach for T1DM treatment. The strong differences between both experimental mouse diabetes models-chemical versus immune response-may explain divergent results. Indeed, MSC transplantation for T1DM treatment is based on their potent anti-inflammatory and/or immunosuppressive properties.

Despite initial experimental transplantation studies with allogeneic MSC suggested they were endowed with strong immune privilege through induction of host tolerance (Barry et al. 2005), more recent studies however indicated that major histocompatibility complex (MHC)-mismatched MSC are relatively much more immunogenic than initially described, inducing significant cell-mediated and humoral immune responses *in vivo* (for review see (Berglund et al. 2017). In line with this evidence, we report here that FVB-derived UC-MSC (H2^q^ MHC haplotype) transplanted within RIP-B7.1 mice (H2^b^ MHC haplotype) were massively targeted by CD11b^high^ peritoneal macrophages, leading to the formation of peritoneal UC-MSC/macrophages aggregates, which were eliminated from the peritoneal cavity through phagocytosis.

Despite their rapid elimination from the host, transplanted UC-MSC however profoundly modified the cellular composition and inflammatory state of resident peritoneal fluid immune cells, by inducing a rapid and massive peritoneal mobilization of CD11b^med^/Ly6C^+/hi^ MDSC, which play a critical role in the resolution of acute inflammation (Saiwai et al. 2013; Li et al. 2021). Additionally and correlating with these findings, UC-MSC transplantation induced a significant upregulation of arginase I (ARG1) expression in PFC, an enzyme with potent immunosuppressive activity and which is highly expressed by murine MDSC and anti-inflammatory M2 type macrophages during the resolution phase of inflammation (Gabrilovich and Nagaraj 2009; Nagaraj et al. 2009; Rath et al. 2014).

Importantly, we also show here that UC-MSC transplantation blunted the expression of the pro-inflammatory marker macrophage inhibitory factor (MIF) in peritoneal fluid cells (PFC), presumably in response to peritoneal MDSC mobilization and induction of the anti-inflammatory response. Although the role of MIF in T1DM development has already been reported (Stosic-Grujicic et al. 2008; Sanchez-Zamora et al. 2016; Korf et al. 2017), none of them have reported an upregulation of MIF in peritoneal immune cells during early diabetes induction. Herein, our finding of upregulated MIF expression in PFC from RIP-B7.1 mice at 8 days of immunization could indicate that peritoneal myeloid cells play an early role in the programmed autoimmune attack, which is in line with previous studies indicating a potential role for resident peritoneal immune cells populations such macrophages and B1-cells in the development of T1DM (Kendall et al. 2004; Silveira and Grey 2006; Emani et al. 2015).

The coincidence of a strong activation of CD11b^hi^ peritoneal macrophages and increased T Cells percentages and Tregs markers expression into PFC of RIP-B7.1 mice at 1 week post-transplantation represents the second temporal wave of immunoregulatory events observed in the peritoneal cavity after the initial target of UC-MSC by peritoneal macrophages and mobilization of MDSC in the peritoneal fluid. It is likely that the mobilization of Tregs within the peritoneal cavity is triggered by the anti-inflammatory/immunosuppressive milieu and T Cells chemokines produced by UC-MSC and MDSC. Mobilization of Tregs in the peritoneal fluid of UC-MSC transplanted IMM-RIP-B7.1 mice likely contributes to EAD attenuation.

The strong upregulation of the antigen-presenting molecules MHC-II onto CD11b^hi^ peritoneal macrophages at 1 week post-transplantation is consistent with their acquisition of a classical activation phenotype as occurring in thioglycollate-elicited or lipopolysaccharide (LPS)-induced peritoneal macrophages (Kim et al. 2015; Pavlou et al. 2017). Such phenomenon could likely indicate that peritoneal macrophages have transitioned into efferocytic or post-phagocytic macrophages presenting UC-MSC-derived antigens to peritoneal fluid T Cells. In support of such premise, MHC-II^hi^ activated peritoneal macrophages displayed a moderate loss of CD11b expression (data not shown), a phenomenon which has been previously reported in post-efferocytic pro-resolving macrophages (Korns et al. 2011; Schif-Zuck et al. 2011).

We also show that UC-MSC transplantation *via* i. p. also induced anti-inflammatory and immunosuppressive responses into secondary lymphoid organs (SLOs) as evidenced by increased percentages of CD11b^+^/Ly6C^+^ MDSC and activated T Cells and Tregs markers in the spleen and pancreatic lymph nodes of transplanted IMM-RIP-B7.1 mice. Of particular interest, splenocytes from early immunized RIP-B7.1 mice were also found to upregulate MIF, evidencing that EAD development is associated with a splenic inflammatory process, a premise consistent with previous reports indicating a role for MIF expression in the development of T1DM and as critical regulator of innate immunity (Calandra and Roger 2003; Stosic-Grujicic et al. 2008; Sanchez-Zamora et al. 2016).

According to their immunoregulatory activity, UC-MSC transplantation led to the upregulation by splenocytes of the anti-inflammatory cytokine IL10 and immunosuppressive enzyme ARG1 whereas the proinflammatory cytokine IFN-γ was downregulated, thus indicating a shift from a pro-to an anti-inflammatory environment. The UC-MSC-mediated upregulation of IL-4 observed in the spleen, is also consistent with the creation of a milieu favorable to the stimulation of activated B Cells and generation of Tregs cells and T helper 2 cells (Cameron et al. 1997; Guo and Rothstein 2013; Wynn 2015; Yang et al. 2017).

Interestingly, the spleen and pancreatic lymph nodes of early UC-MSC transplanted mice display a transient increase in the number of activated CD19^+^/CD25^+^ B Cells, which is the subset of B Cells containing regulatory B Cells (Bregs), (Kessel et al. 2012; de Andres et al. 2014; Hong et al. 2019; Ben Nasr et al. 2021). Interestingly, different studies already indicated how MSC transplantation induced increased Bregs, which exert immunosuppression through secretion of IL-10 (Kleffel et al. 2015; Cho et al. 2017; Luk et al. 2017; Phillips et al. 2017; Phillips et al. 2019; Qu et al. 2021). Therefore, the observed high upregulation of IL-10 expression in the spleen of UC-MSC transplanted IMM RIP-B7.1 mice is consistent with a higher number of Bregs in response to UC-MSC transplantation.

Interestingly, UC-MSC transplantation in IMM-RIP-B7.1 mice also exhibited an increase percentage of CD11b^+^/Ly6C^+^ MDSC and CD25^+^/CD4^+^ activated T Cells in pancreatic lymph nodes (PLN), which together with increased IL-4 and FoxP3 mRNA expression could indicate an expansion of Tregs.

We also show that UC-MSC transplantation significantly reduced pancreatic infiltration of CD4^+^ and CD8^+^ T, CD19^+^ B and CD11b^+^ myeloid cells in IMM-RIP-B7.1 mice. Myeloid cells infiltrated in the pancreas of IMM-RIP-B7.1 mice were accordingly identified as [F4/80^+^ CD206^-^] M1 pro-inflammatory macrophages, which are a major cellular effector in insulitis (Korf et al. 2017).

Of special interest, we also found that hyperglycemic IMM-RIP-B7.1 mice also displayed increased pancreatic infiltration of [CD11b^+^ MHC-II^-^ Gr1^+^] MDSC, which is very striking due to their opposite role with M1 pro-inflammatory macrophages and also since a previous study reported decreased MDSC in islets of NOD mice (Fu et al. 2012). Our results are however supported by other studies showing increased MDSC in the peripheral blood and secondary lymphoid organs of NOD and STZ mice models as well as in T1DM and T2DM patients (for review see (Wang et al. 2021). A higher number of MDSC during insulitis development is likely a compensatory mechanism to suppress diabetogenic T Cell proliferation.

Overall, our study provides evidence of multiple immunomodulary responses induced by UC-MSC in the RIP-B7.1 rodent model of T1DM, inducing a strong delay of their EAD. Whether the treatment with a second dose of UC-MSC would be enough to postpone the resurgence of T1DM is also an issue that would require additional studies. Our results therefore support the approach of the i. p. transplantation of allogenic UC-MSC for the treatment of early-diagnosed human T1DM patients.

## Materials and methods

### Isolation and culture of mouse UC-MSC

UC-MSC were isolated from E17 old FVB (H2^q^) mice fetuses. Guidelines for the animal research protocols were established and approved by the Animal Experimentation and Ethics Committee of CABIMER (CEEA-CABIMER). Umbilical cords (UC) were sequentially trypsinized into PBS +2% bovine serum albumin (BSA) containing first 0.25% trypsin (Gibco) to detach outermost epithelial lining cells. Partially digested UC were then digested and then 2 mg/mL type Ia collagenase (Sigma) to obtain UC-MSC. Both digestions were done in a water bath (37°C, 15 min) under agitation. Umbilical cords stromal cells were seeded at 20.000 cells/cm^2^ into 140 mm Petri dishes (Nunc) and cultured into a DMEM, low glucose, GlutaMAX™, pyruvate medium (GIBCO) supplemented with 5% FBS, 1% antibiotics, 1X MEM non-essential amino acids (Gibco), 1X insulin transferrin selenium (ITS), 100 µm 2-mercaptoethanol, 10 ng/mL bFGF and 10 ng/mL EGF in an incubator under low oxygen pressure for 72 h (5% CO2, 3% O_2_, 37°C). UC-MSC were harvested and frozen in multiple aliquots. Prior transplantation, UC-MSC were thawed and subcultured for 72 h in the same media.

### RIP-B7.1 mice

Mice experimentations were approved by the CABIMER Animal Committee, and performed in accordance with the Spanish law on animal use RD 53/2013. Mice were housed under specific pathogen-free conditions in the animal facility of CABIMER. Experimental autoimmune diabetes (EAD) was induced in 8–9 weeks old RIP-B7.1 mice transgenic mice (C57BL/6 H-2^b^ background) by injection of 50 μg preproinsulin (ppINS) expression plasmid (PlasmidFactory GmbH, Germany) into each anterior tibialis muscles (Cobo-Vuilleumier et al. 2018). One week later, mice were intraperitoneally injected with 300 µL of DMEM (vehicle) or DMEM containing 5 × 10^5^ UC-MSC under anesthesia using the IVIS system. Mice were euthanized at 1 day, 1 and 7 weeks after transplantation. Peritoneal fluid cells, splenocytes, pancreatic lymph nodes cells and pancreatic stromal cells were collected for FC and quantitative PCR analysis.

### Glycemic records

Circulating glucose levels were measured weekly from tail vein blood samples using an Optium Xceed glucometer (Abbott Scientifica SA, Barcelona, Spain). Two consecutive measurements of non-fasting blood glucose ≥250 mg/dL were considered to indicate overt diabetes.

### UC-MSC tracking and biodistribution analysis

Cultured UC-MSC suspensions were fluorescently labelled for 10 min with 10 µM carboxyfluorescein succinimidyl ester (CFSE) or 320 μg/mL XenoLight DiR (PerkinElmer) in DMEM for 20 min for *in vivo* biodistribution analysis using IVIS^®^ Spectrum *in vivo* imaging system (PerkinElmer). Fluorescently labelled UC-MSC cultures were extensively washed before harvesting with trypsin. Fluorescently labelled UC-MSC suspension were identically washed twice through centrifugation before transplantation.

### ELISA: Plasmatic cytokines measurements

Blood samples were collected by cardiac puncture and centrifuged into Microvette CB 300 K2E, Sarstedt (Nümbrecht, Germany) with EDTA and plasma collected and aliquots frozen at −80°C. Cytokine levels were determined using the mouse V-PLEX ProInflammatory Panel 1 kit 10-Plex (Meso Scale Discovery, Rockville, USA) and data acquired on an MSD MESOTM QuickPlex SQ120.

### Pancreas histology and immunohistochemistry

Pancreases sections were obtained as previously reported (Lorenzo et al. 2015). Antibodies used in this study are listed in Supplementary Tables S1, S2. Nuclei were counterstained with DAPI, and sections mounted with DAKO fluorescent mounting medium. Images were acquired using either Leica DM6000B or a Leica TCS SP5 confocal microscope. Insulitis was scored in H&E stained paraffin sections of pancreas, as previously described (Mellado-Gil et al. 2016). For insulitis score, a total of 3 mice for each experimental group were analyzed at 7 weeks after transplantation. Total number of islets analyzed per group was 132 for CT, 82 for CT+5.10^5^ UC-MSC, 71 for IMM, 183 for IMM+2.10^5^ UC-MSC, 177 for IMM+5.10^5^ UC-MSC and 180 for IMM+2 doses of 2.10^5^ UC-MSC.

### Peritoneal drainages, spleen, pancreatic lymph nodes and pancreas sampling

Peritoneal drainages were collected with minor modifications as previously described (Ray and Dittel 2010). Briefly, skin of the abdominal wall was removed and the exposed abdominal wall gently cleaned with ethanol prior injecting 8-10 mL cold PBS solution containing 2% BSA and 2.5 mM EDTA into the peritoneal cavity. After a quick gentle massage, mice were placed side down on top of a platform and a small incision in the abdominal wall was made to collect peritoneal lavages into non-adherent Petri dishes placed on ice. Spleen and pancreases were isolated and stored into cold PBS+2%BSA+2.5mMEDTA solution on ice. Pancreatic lymph nodes were surgically removed and collected under magnification. Splenocytes and pancreatic lymph nodes cells were teased into single cell suspensions by gentle disruption between the frosted ends of slides using a cold PBS+2% BSA+2.5 mM EDTA solution and filtered using 100 μm cell strainer (BD Falcon). See also Supplementary Methods for more detailed information.

### Flow cytometry

Aliquots of freshly isolated cells resuspended in cold PBS+2%BSA+2.5mMEDTA solution were incubated with antibodies for 30 min on ice. Stained cells were then fixed with 4% paraformaldehyde and then analyzed on a FACSCalibur flow cytometer (BD Biosciences, Madrid, Spain). Data were analyzed using Flowing Software version 2.5.1 (Turku Centre for Biotechnology, Univ. Turku, Finland).

### RNA isolation and quantitative PCR (QPCR)

RNA isolation and QPCR was performed as previously reported (Lachaud et al., 2014). Briefly, total RNA content was extracted with Trizol reagent (Invitrogen), and total RNA was reverse-transcribed into cDNA by using MMLV reverse transcriptase (Promega, Madison, WI, United States). qPCR was performed using SYBR-Green and detected using an ABI Prism 7500 system (Applied Biosystems, Foster City, CA, United States). Quantification of the mRNA level of each gene was normalized to β-actin mRNA. Results show folds of mRNA expression of a given gene relative to its expression in control non-immunized RIP-B7.1 mice, which served as the calibrator sample (set as 1). Primers sequences are listed in Supplementary Table S1. Primers aliquots can be obtained upon request.

### Figures edition

Graphs were created using GraphPad Prism 7.00. Figures were created using Adobe Photoshop.

### Statistical analysis

Results are expressed as mean ± s. e.m. N, is the number of mice analyzed for each group, in each analysis is ≥5 for flow cytometry and is ≥4 for qPCR. Statistical analysis was performed using the GraphPad Prism 7.00 Software (GraphPad, La Jolla, United States). Statistical differences were estimated by one-way ANOVA or Student´s test, whichever was appropriate.

## Data Availability

The raw data supporting the conclusion of this article will be made available by the authors, without undue reservation.

## Abbreviations

ARG1, Arginase 1; Breg, B regulatory cells; CFSE, Carboxyfluorescein succinimidyl ester; EAD, Experimental autoimmune diabetes; FC, Flow cytometry; H&E, Hematoxylin and eosin; i.p., Intraperitoneal; IL-10, Interleukin 10; IL-4, Interleukin 4; IMC, Immature myeloid cells; iNOS, Inducible nitric oxide synthase; MDSC, Myeloid-derived suppressor cells; MHC-II, Major histocompatibility complex 2; MIF, Macrophage inhibitory factor; NOD, Non-obese diabetic mice; PDGR-β, Platelet derived growth factor beta; PFC, Peritoneal fluid cells; PLN, Pancreatic lymph nodes; ppINS, Preproinsulin; RIP-B7.1, Rat insulin promoter-B7.1; SLOs, Secondary lymphoid organs; T1DM, Type 1 diabetes mellitus; Treg, Regulatory T Cells; UC-MSC, Umbilical cord mesenchymal stromal cells.
