# Supplementary figures and images for: Umbilical cord mesenchymal stromal cells transplantation delays the onset of hyperglycemia in the RIP-B7.1 mouse model of experimental autoimmune diabetes through multiple immunosuppressive and anti-inflammatory responses
Source: Front Cell Dev Biol. 2023 Feb 15;11:1089817. doi: 10.3389/fcell.2023.1089817 (PMC9976335; doi:10.3389/fcell.2023.1089817)

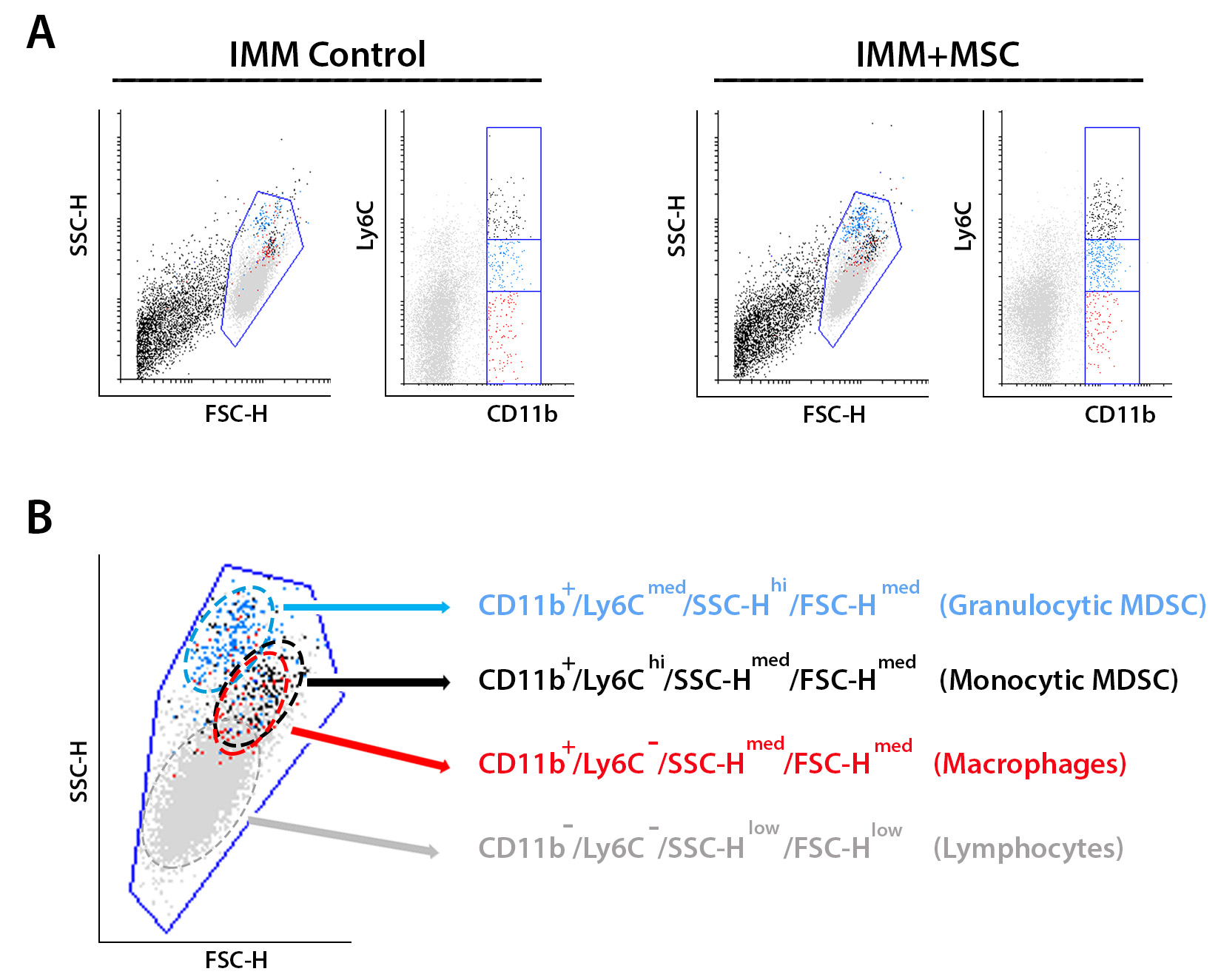

Supplement: Supplementary file 1 [file Image3.JPEG]

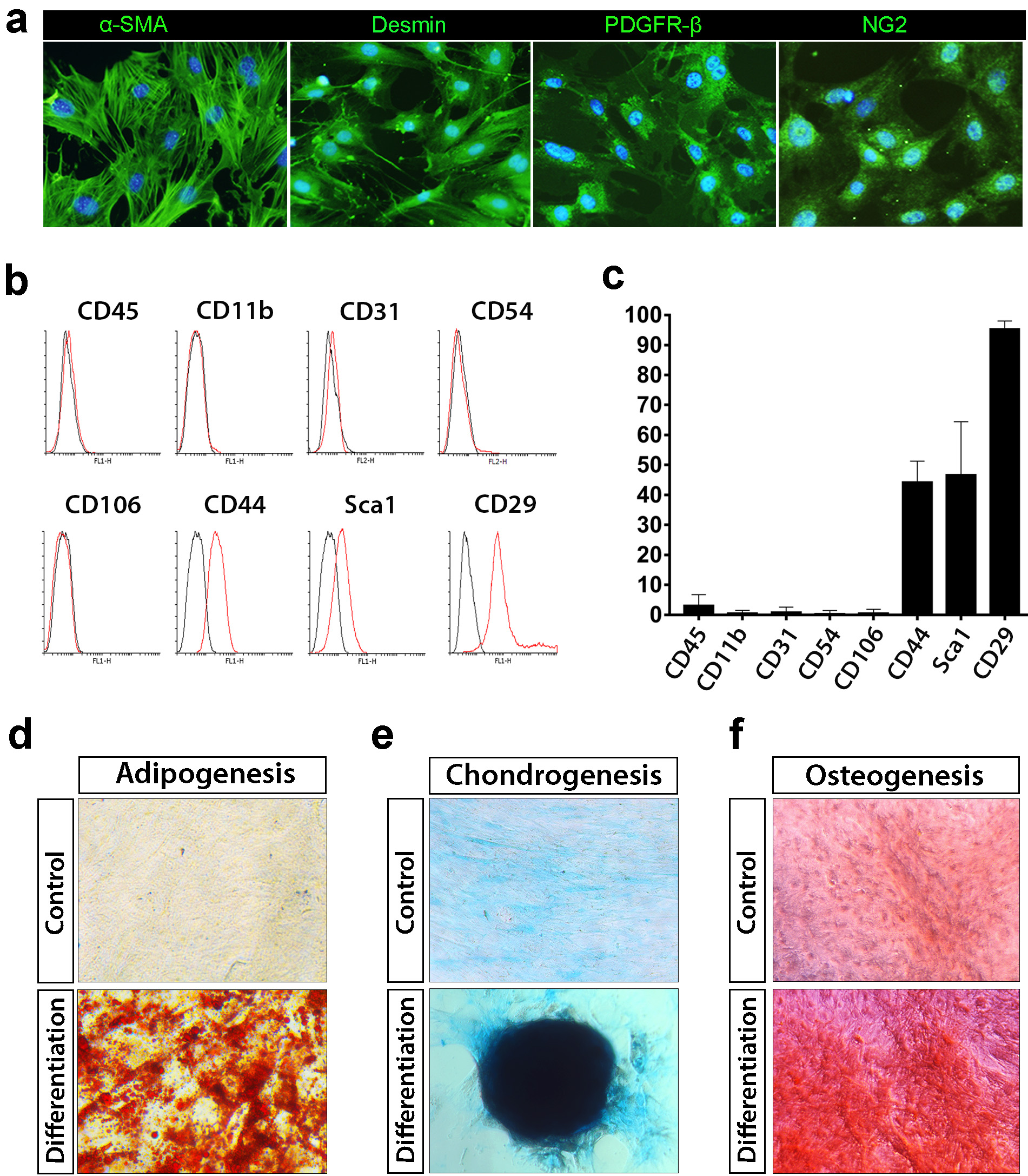

Supplement: Supplementary file 2 [file Image1.JPEG]

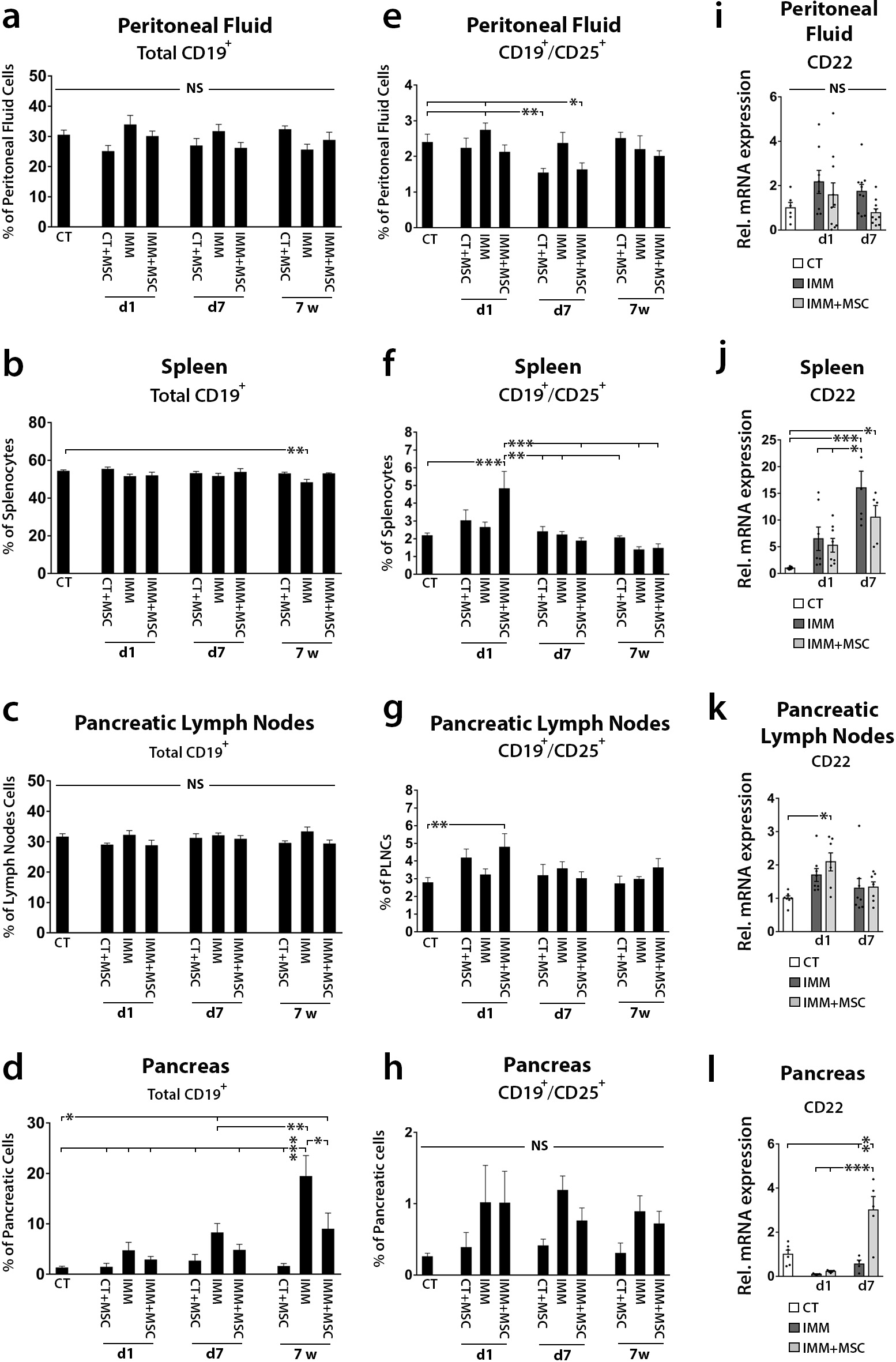

Supplement: Supplementary file 3 [file Image4.JPEG]

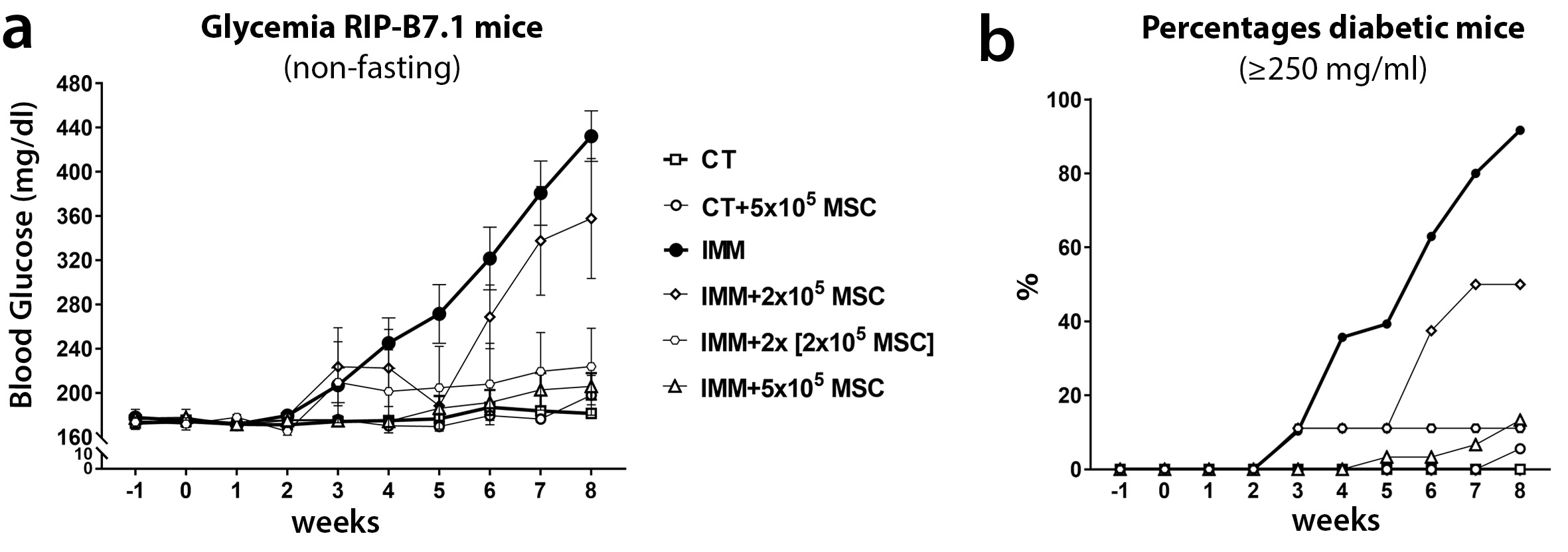

Supplement: Supplementary file 4 [file Image2.JPEG]

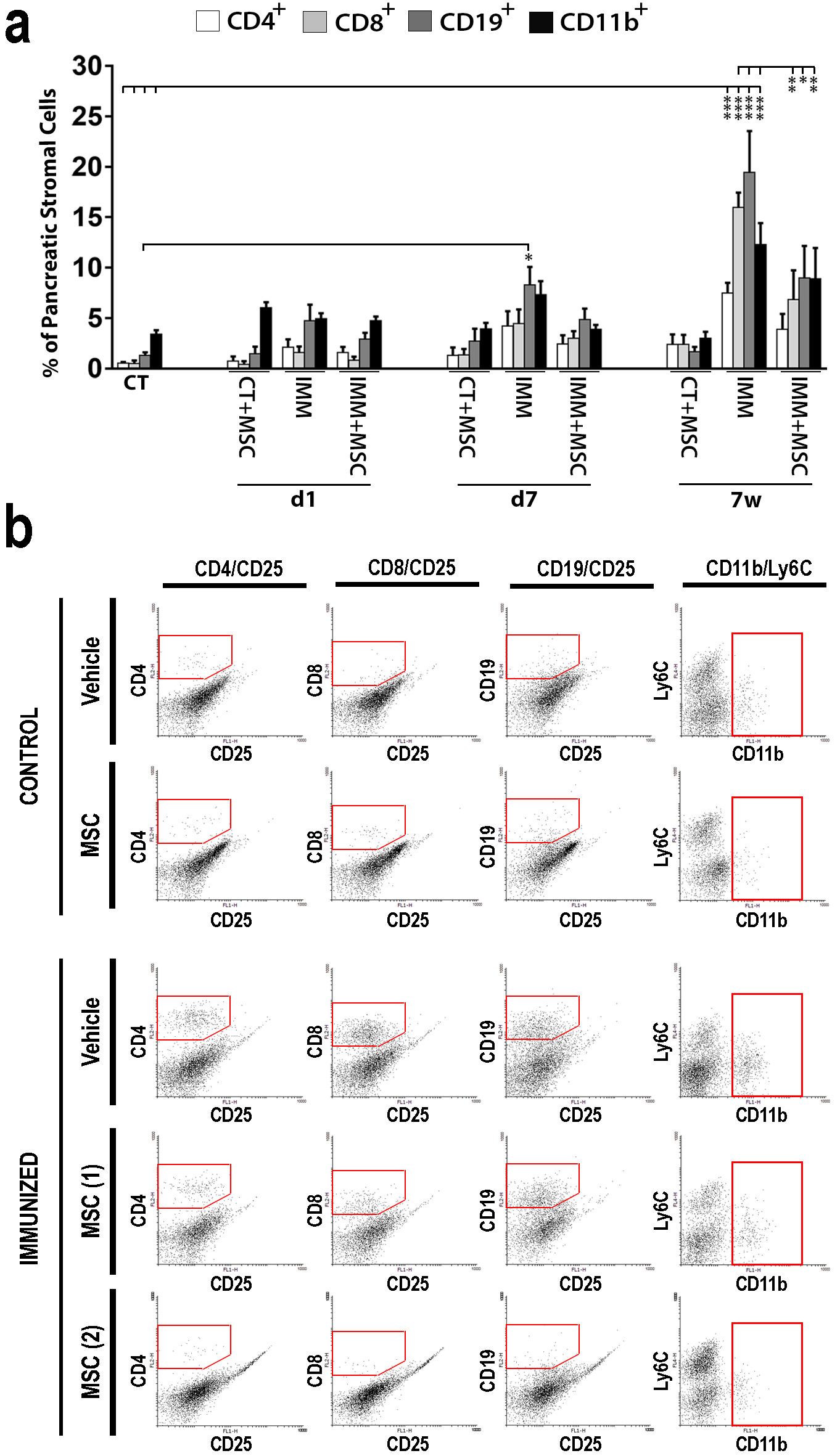

Supplement: Supplementary file 5 [file Image5.JPEG]
