# Supplementary material for: Umbilical cord mesenchymal stromal cells transplantation delays the onset of hyperglycemia in the RIP-B7.1 mouse model of experimental autoimmune diabetes through multiple immunosuppressive and anti-inflammatory responses
Source: Front Cell Dev Biol. 2023 Feb 15;11:1089817. doi: 10.3389/fcell.2023.1089817 (PMC9976335; doi:10.3389/fcell.2023.1089817)
Supplement: Supplementary file 6 [file DataSheet1.pdf]

SUPPLEMENTARY FIGURES

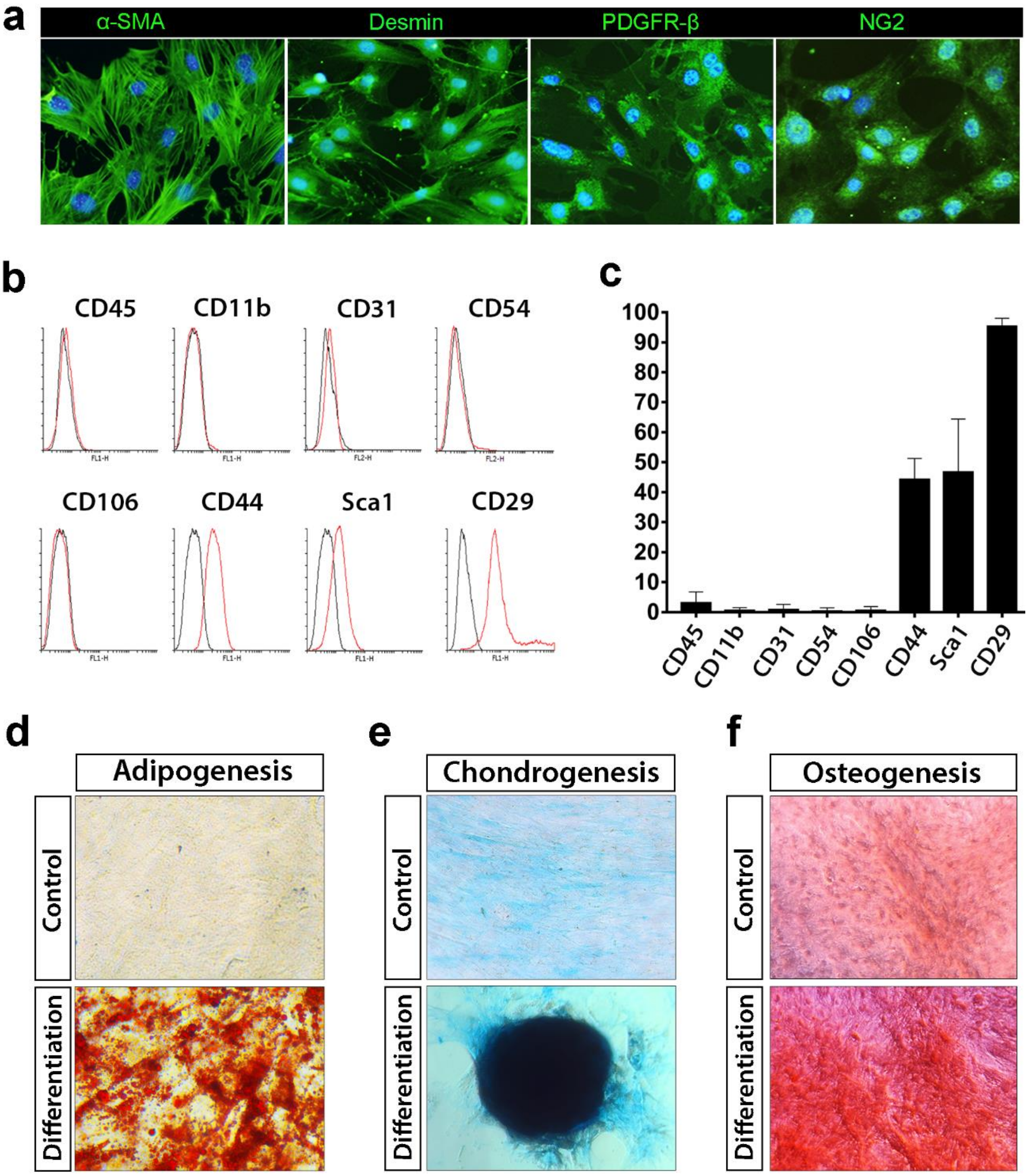

SUPPLEMENTARY FIGURE 1

Supplementary Figure 1. Cultured UC-MSC express pericytes and mesenchymal stromal cells markers and display mesodermal multipotent differentiation capacity. (**a**) Representative immunofluorescence stainings of subcultured UC-MSC against α-SMA, Desmin PDGFR-β and NG2, markers expressed by pericytes and MSC. Scale bar is 50 µm. (**b**) Shows representative flow cytometry expression histograms of subcultured UC-MSC against different cell-surface markers, showing expression of the murine mesenchymal stromal cells (MSC) markers CD44, Sca1 and CD29. (**c**), Summary quantification of cell-surface markers expression into subcultured UC-MSC (values are means ± s.e.m. of n=5 independent cultures). (**d**-**f**), Mesodermal multi-differentiation assay of UC-MSC cultured for 2 weeks under adipogenic (**d**), chondrogenic (**e**) and osteogenic (**f**) control media (upper) or inductive media (lower) and respectively stained for detecting lipid droplets with oil red, glycosaminoglycans with alcian blue and calcium deposits with alizarin red.

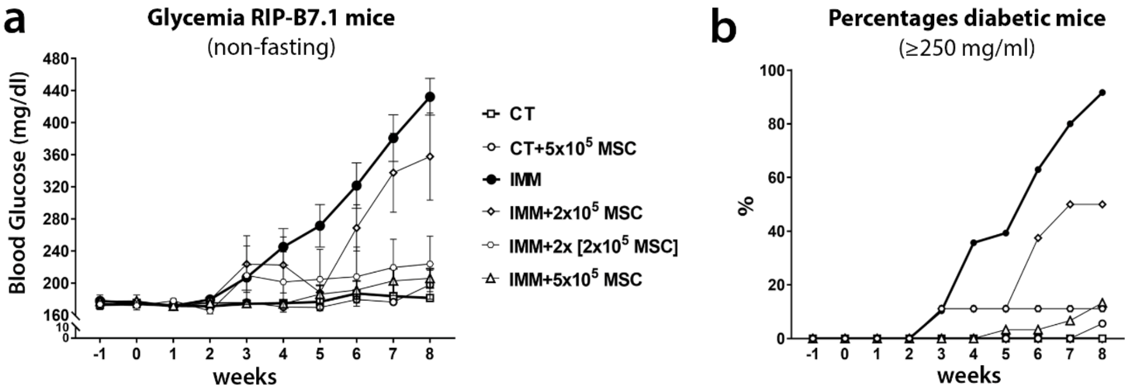

SUPPLEMENTARY FIGURE 2

Supplementary Figure 2. UC-MSC transplantation dose-dependently reduces EAD incidence. (**a**) Summary of non-fasting blood glucose measurements in control (CT) and immunized (IMM) RIP-B7.1 mice, transplanted with vehicle or different doses of UC-MSC. (**b**) Mean percentages of diabetic RIP-B7.1 mice (≥250 mg/dl) from the different experimental groups. (**a**-**b**) CT, n=18; CT+5x10^5 UC-MSC, n=18; IMN, n=28; IMM+2x10^5 UC-MSC, n=9; IMM+5x10^5 UC-MSC, n=31; IMM+ 2 doses of 2x10^5 UC-MSC, n=9.

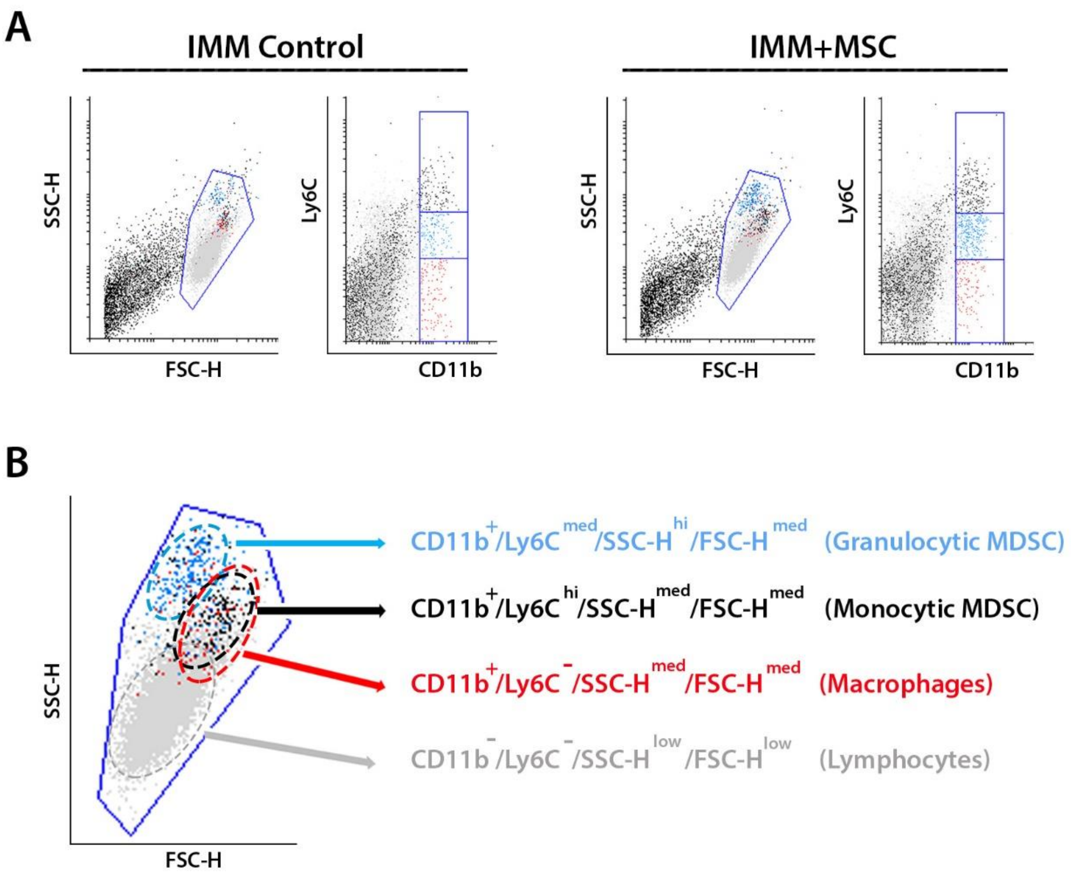

SUPPLEMENTARY FIGURE 3

Supplementary Figure 3. **Flow cytometric identification of splenic myeloid cells subsets in IMM-RIP-B7.1 mice not transplanted (IMM control) and transplanted with UC-MSC (IMM+MSC)**. (**a**) Left, shows representative SSC-H/FSC-H and Ly6C/CD11b dot plots of splenocytes from IMM-RIP-B7.1 mice control (left panel) and transplanted with UC-MSC at 7 days after transplantation. Splenocytes were gated on the basis of their SSC-H and FSC-H characteristics (blue gate in SSC-H/FSC-H dot plots). Right dot plots in panels show CD11b and Ly6C coexpression levels of R1 gated splenocytes. CD11b^{+} were divided in 3 subpopulations based on their differential expression levels of Ly6C. (**b**) Shows SSC-H and FSC-H subpopulations analysis in splenocytes from IMM-RIP-B7.1 mice at 7 days after UC-MSC transplantation. Subpopulations could be identified as corresponding to CD11b^{+}/Ly6C^{-} macrophages (red dots), CD11b^{+}/Ly6C^{med} granulocytic MDSC (blue dots) and CD11b^{+}/Ly6C^{high} monocytic MDSC (dark dots), distinguishable in size and complexity from splenic lymphocytes (grey dots).

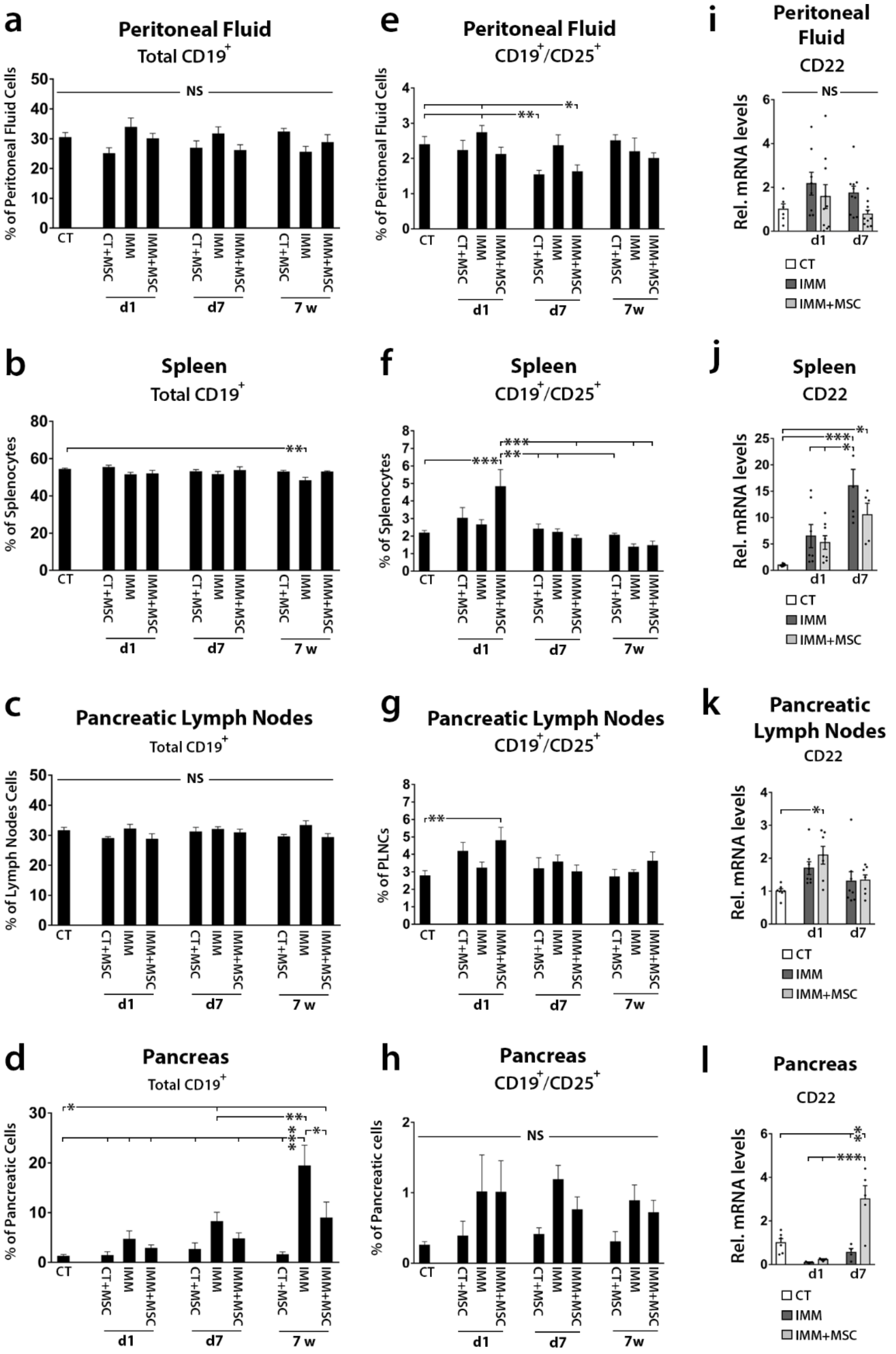

SUPPLEMENTARY FIGURE 4

Supplementary Figure 4. **UC-MSC transplantation increases transiently activated B cells in the spleen and pancreatic lymph nodes of IMM-RIP-B7.1 mice and lowers by contrast pancreatic infiltrated B cells**. (**a**-**d**) Flow cytometric quantification of total $CD19^+$ B cells percentages within peritoneal fluid cells (**a**), splenocytes (**b**), pancreatic lymph nodes cells (**c**) and pancreatic stromal cells (**d**). (**e**-**h**) Flow cytometric quantification of $CD19^+$/$CD25^+$ activated B cells percentages within peritoneal fluid cells (**e**), splenocytes (**f**), pancreatic lymph nodes cells (**g**) and pancreatic stromal cells (**h**). (**a**-**h**) Cells were collected from CT and IMM-RIP-B7.1 mice, at 24h, 7 days and 7 weeks post-transplantation with vehicle or $5x10^5$ UC-MSC. (**i**-**l**) Q-PCR analysis of CD22 mRNA expression in peritoneal fluid cells (**i**), splenocytes (**j**), pancreatic lymph nodes cells (**k**) and pancreatic stromal cells (**l**) collected from control (CT) or immunized (IMM) RIP-B7.1 mice transplanted with vehicle and $5x10^5$ UC-MSC at day 1 and 7 post-transplantation. Results show relative mRNA expression to CT mice (value set as 1). (**a**-**l**) Values are mean ± s.e.m of n≥5 mice for each experimental group. ***, *P*≤0.01; **, *P*≤0.03; *, *P*≤0.05, one-way ANOVA. NS is not significant.

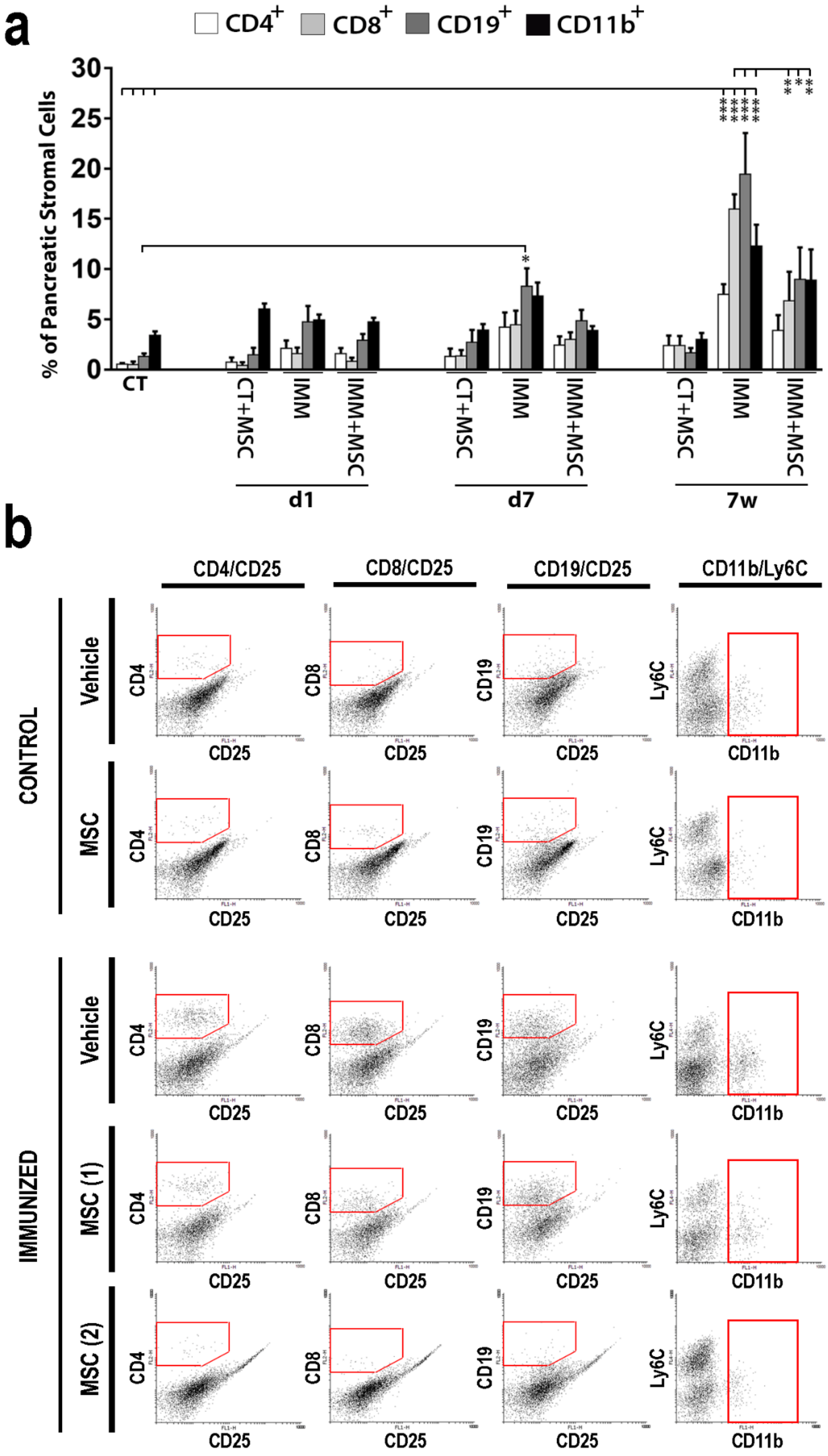
a
CD4+
CD8+
CD19+
CD11b+
% of Pancreatic Stromal Cells
0
5
10
15
20
25
30
CT
CT+MSC
IMM
IMM+MSC
d1
d7
7w
b
CD4/CD25
CD8/CD25
CD19/CD25
CD11b/Ly6C
CONTROL
Vehicle
MSC
IMMUNIZED
MSC (1)
MSC (2)
CD4
CD8
CD19
Ly6C
CD25
CD11b

SUPPLEMENTARY FIGURE 5

Supplementary Figure 5. **UC-MSCs transplantation significantly reduces pancreatic leukocytes infiltration in IMM-RIP-B7.1 mice**. (**a**) Shows summary flow cytometric quantification of percentages of $CD4^+$ and $CD8^+$ T cells, $CD19^+$ B cells and $CD11b^+$ myeloid cells within pancreatic stromal cells from CT- or IMM-RIP-B7.1 mice at 1 day (d1), 7 days (d7) and 7 weeks (7w) after vehicle or $5x10^5$ UC-MSCs transplantation. Values are mean ± s.e.m of n≥5 mice for each experimental group. ***, P≤0.01; **, P≤0.03; *, P≤0.05, one-way ANOVA. (**b**) Shows representative CD4/CD25, CD8/CD25, CD19/CD25 and CD11b/Ly6C dot plots expression for control or immunized RIPB7.1 mice at 7 weeks post transplantation with UC-MSC or vehicle, which were used to quantify percentages of $CD4^+$, $CD8^+$, $CD19^+$ and $CD11b^+$. Red gates localize $CD4^+$, $CD8^+$, $CD19^+$ lymphocytes and $CD11b^+$ myeloid cells. IMM+MSC (1) and IMM+MSC (2) correspond to two distinct immunized RIPB7.1 mice transplanted with UC-MSC and that show distinct levels of lymphocytes and myeloid cells infiltration, being high for IMM+MSC (1) and low for IMM+MSC (2).

Supplementary Material and Methods:

Peritoneal drainages

For peritoneal drainages collections, mice were killed by cervical dislocation and abdominal skin was removed with the use of scissors. The exposed abdominal wall was then gently sterilized with ethanol and dried. Mice were then fixed side down on the border of a platform. An untreated P80 sterile Petri dish was placed on ice at a distance of 10 centimeters below the mouse. A total volume of 8-10 ml cold PBS solution containing 2% BSA and 2.5 mM EDTA was then injected into the peritoneal cavity with the use of a 21G needle. Special care was taken to not damage visceral organs during injection of the PBS solution. A gentle massage of the abdomen was done just before performing a small incision in the center of abdominal wall. PBS solution containing peritoneal immune cells was then gently collected in the Petri dish. The use of EDTA and non-adherent petri dish to collect peritoneal lavage cells was found to be of critical importance to avoid adherence of peritoneal macrophages. PBS solution containing peritoneal lavage cells was then directly transferred to 15 ml polystyrene conical tube on ice.

Splenocytes isolation

Spleen were surgically removed from the sacrificed mice just after peritoneal drainages and were transferred into cold PBS+2%BSA+2.5mMEDTA solution on ice. Spleens were then placed between the frosted ends of two glass slides and mechanically disrupted with constant resuspension of the cells in the cold PBS solution. The solution of splenocytes was then filtered through a 100 µm cell strainer (BD Falcon) placed into a 50ml conical tube on ice and the resulting cell

suspension was then centrifuged at 4ºC and cells subjected to erythrocytes lysis. The resulting splenocytes pellet was finally resuspended into 1 ml of cold PBS+2%BSA+2.5mMEDTA solution and aliquots used for labelings with conjugated antibodies for flow cytometry analysis or for qPCR analysis.

Pancreatic lymph nodes isolation

Pancreases were then surgically removed together with the mesentery and placed into a Petri dish containing cold PBS+2%BSA+2.5mMEDTA solution on ice. The connected pancreas and mesentery were then surgically separated under magnification and with the use of a small scissor and precision tweezers, pancreatic lymph nodes were removed and transferred into a clean Petri dish placed on ice and containing cold PBS+2%BSA+2.5mMEDTA solution. The isolation of pancreatic lymph nodes cells was then performed almost identically as described previously for splenocytes isolation, with the use of the frosted ends of glass slides. Pancreatic lymph nodes cells were not subjected to erythrocytes lysis and directly centrifuged at 4ºC, prior being used for antibodies labelings or RNA extraction for qPCR analysis.

Pancreatic stromal cells isolation

Following the collection of pancreatic lymph nodes, pancreases were washed in cold PBS checked again under magnification for ensuring total removal of pancreatic lymph nodes. Pancreases were then transferred to 15 ml conical polystyrene tube containing 3 ml pre-warmed DMEM low glucose basal containing 2 mg/ml collagenase type I (Sigma-Aldrich C0130) and 1 mg/ml collagenase type V (Sigma-Aldrich C9263) + 1%BSA. Digestion samples were

gently shaken 2 to 3 times during the digestion procedure that usually last 15 to 20 minutes. Digested pancreases were then filtered onto 100 µm cell strainer (BD Falcon) to collect the resulting cell suspensions that was finally centrifuged and resuspended into cold PBS+2%BSA+2.5mMEDTA solution and placed on ice until use for antibodies labelling or extraction of RNA.

Supplementary Table 1: Primary antibodies Fluorescent conjugated antibodies

REAGENT	SOURCE	REFERENCE	DILUTION
Mouse monoclonal anti-αSMA	Sigma-Aldrich	A5228	1:300
Mouse monoclonal anti-SM22α	Abcam	ab28811	1:200
Rabbit sera anti-Desmin	Sigma-Aldrich	D8281	1:100
Rabbit polyclonal anti-Smoothelin B	Santa Cruz	sc-28562	1:200
Rabbit polyclonal anti-PDGFR-β	Santa Cruz	sc-432	1:200
Rabbit polyclonal anti-NG2	Santa Cruz	sc-20162	1:100
Rabbit anti-insulin	Santa Cruz	sc-91168	1:200
Mouse anti-insulin	Sigma-Aldrich	I2018	1:250
Rabbit anti-glucagon	Cell Signalling	2760	1:200
Mouse anti-glucagon	Sigma-Aldrich	A944	1:150
XenoLight DiR NIR Fluorescent Dye	PerkinElmer	125964	25µg/ml
5(6)-CFDA, CE	ANASPEC	AS-89000	10µM
Fluoresbrite 0,75 µm Microspheres	Polysciences	07766	10^9/ml

Supplementary Table 2: Fluorescent conjugated antibodies

REAGENT	SOURCE	REFERENCE	DILUTION
FITC rat anti-mouse CD45	BD	553080	1:100
FITC rat anti-mouse CD106	BD	553332	1:100
FITC rat anti-mouse CD44	BD	553133	1:100
FITC rat anti-mouse Sca1	BD	553335	1:100
FITC rat anti-mouse CD29	BD	555005	1:100
PE rat anti-mouse CD31	BD	553373	1:100
PE rat anti-mouse CD54	BD	553253	1:100
PE rat anti-mouse CD4	BD	553048	1.100
PE rat anti-mouse CD8	BD	553032	1.100
PE rat anti-mouseCD19	BD	561736	1.100
BB515 rat anti-mouse CD25	BD	564424	1.100
FITC rat anti-mouse CD11b	BD	557396	1:100
PE rat anti-mouse CD11b	BD	553311	1.100
PE rat anti-mouse Gr1	BD	553128	1:100
APC-Cy™7 rat anti-mouse Ly6C	BD	560596	1:100
PE rat anti-mouse I-A/I-E	BD	562010	1.100
AF647 rat anti-mouse I-A/I-E	BD	562367	1.100
PE rat anti-mouse CD86	BD	561963	1.100
FITC rat IgG2b, k isotype control	BD	553988	1.100
FITC hamster IgM, λ isotype control	BD	553960	1.100
PE hamster IgG1, k isotype control	BD	553972	1.100
PE rat IgG2a, k isotype control	BD	553390	1.100
PE anti-mouse CD206 (MMR)	Biolegend	141705	1.100
APC anti-mouse F4/80 Antibody	Biolegend	123116	1.100
Alexa Fluor® goat anti-rabbit IgG	Invitrogen™	A-11008	1.200
Alexa Fluor® goat anti-mouse IgG	Invitrogen™	A-11001	1.200

Supplementary Table 3: Primers

GENE	Forward	Reverse
**B7.1-INS1**	CAAACAACAGCCTTACCTTCGG	GCCTCCAAAACCTACACATCCT
**MIF**	CAGAACCGCAACTACAGTAAG	GCAGCGTTCATGTCGTAAT
**IFN-γ**	TCAGCAACAGCAAGGCGAAA	TCTCTTCCCCACACCCGAATC
**IL-10**	GCTCTTACTGACTGGCATGAG	CGCAGCTCTAGGAGCATGTG
**ARG1**	CTCCAAGCCAAAGTCCTTAGAG	AGGAGCTGTCATTAGGGACATC
**CD4**	AGGTGATGGGACCTACCTCTC	GGGGCCACCACTTGAACTAC
**IL-4**	GGTCTCAACCCCCAGCTAGT	CCCTTCTCCTGTGACCTCGT
**FoxP3**	GGCCCTTCTCCAGGACAGA	GCTGATCATGGCTGGGTTGT
**CD22**	ATGCGCGTCCATTACCTGTG	TCAACGGTCCAATCATTTGCT
**β-actin**	TCCTGTGGCATCCACGAAACTACA	ACCAGACAGCACTGTGTTGGCATA

Abbreviations: IFN-γ; Interferon gamma; ARG1, Arginase 1; IL-4, Interleukin-4; IL-10, Interleukin-10; INS1, Insulin 1; MIF, Macrophage Inhibitory Factor.
